# Identification of a key smooth muscle cell subset driving ischemic cardiomyopathy progression through single-cell RNA sequencing

**DOI:** 10.1038/s41598-025-09928-6

**Published:** 2025-07-27

**Authors:** Wenyang Nie, Yong Wang, Yuanyuan Xiao, Zhiheng Lin, Jingwen Zhang, Zhijie Zhao, Zhen Wang

**Affiliations:** 1 First Clinical Medical College, Shandong University of Traditional Chinese Medicine, 16369 Jingshi Rd, Jinan, 250014 China; 2 Department of Cardiovascular Diseases, Affiliated Hospital of Shandong University of Traditional Chinese Medicine, 16369 Jingshi Rd, Jinan, 250014 China; 3 Department of Plastic and Reconstructive Surgery, Shanghai Ninth People’s Hospital, School of Medicine, Shanghai Jiao Tong University, 639 Zhi Zao Ju Rd, Shanghai, 200011 China; 4 Department of Ophthalmology, Shanghai University of Medicine & Health Sciences Affiliated Zhoupu Hospital, 1500 Zhouyuan Rd, Shanghai, 201318 China; 5 Department of Gynecology, Longhua Hospital, Shanghai University of Traditional Chinese Medicine, Shanghai, 200032 China

**Keywords:** Single-cell RNA sequencing, Cardiomyopathy, Smooth muscle cells, Ferroptosis, Exosomes, Inflammation, Cytokines, Immunochemistry, Ion channels, Metabolomics, Proteins, Structural biology, Bioinformatics, Cytological techniques, Immunological techniques, Metabolomics, Proteomic analysis, Sequencing, Cell adhesion, Cell death, Cell division, Cell growth, Cell migration, Cell signalling, Mechanisms of disease, Senescence, Cardiovascular biology, Biological techniques, Cell biology, Immunology, Physiology, Stem cells, Anatomy, Biomarkers, Cardiology, Health care, Medical research, Pathogenesis, Risk factors

## Abstract

Cardiomyopathy encompasses a range of diseases that severely affect the complex functions of the heart, involving structural and functional abnormalities, and is associated with high mortality. Recent studies have highlighted the critical role of ferroptosis in regulating oxidative stress and inflammation in cardiomyopathy. In this study, we established that the C6 S100A4+ SMCs subpopulation is critical by performing an integrated single-cell analysis of the known publicly available data GSE145154. We validated the role of S100A4 in SMCs through in vitro experiments, providing evidence for its potential as a therapeutic target. Furthermore, these cells interact with endothelial cells through the PTN-NCL pathway, influencing disease progression. Key transcription factors, including KLF2, FOS, FOSB, and JUNB, were identified. This key subpopulation, along with its associated signaling pathways, marker genes, stemness genes, and transcription factors, may offer new insights for preventing the onset and progression of cardiomyopathy, particularly ischemic cardiomyopathy.

## Introduction

Cardiomyopathy refers to a group of diseases characterized by diverse structural and functional changes in the heart, which often result in poor prognosis and a high risk of mortality^1,2^. According to the classification system proposed by the American Heart Association (AHA) in the early twenty-first century, cardiomyopathies are divided into primary and secondary types. Primary categories include dilated, hypertrophic, and restrictive cardiomyopathies, while secondary causes encompass various etiologies such as ischemic, metabolic, infectious, toxic, autoimmune, and neuromuscular factors^1^. Dilated cardiomyopathy (DCM) is the most common form of non-ischemic cardiomyopathy and the most frequently encountered subtype overall^3,4^. Restrictive cardiomyopathy (RM) comprises a heterogeneous group of disorders characterized by persistent restrictive pathophysiology, diastolic dysfunction, non-dilated ventricles, and atrial enlargement. These features persist regardless of ventricular wall thickness or systolic function, highlighting the diverse nature of RM^5^. Ischemic cardiomyopathy (ICM) is the most prevalent type of cardiomyopathy, primarily resulting from coronary atherosclerosis. Obstruction of blood flow due to atherosclerosis leads to myocardial hypoxia and subsequent cell death, triggering cardiac remodeling, including fibrosis and scar formation. Over time, the cumulative effects of hypoxia and fibrosis impair myocardial contractility, leading to cardiac hypertrophy, dilation, and, eventually, heart failure^6,7^. Treatment of ICM primarily targets the underlying cause, with anatomical revascularization being the preferred method to address coronary atherosclerosis. Pharmacological therapies focus on managing risk factors for atherosclerosis, while surgical approaches such as coronary artery bypass grafting (CABG), endarterectomy, and percutaneous coronary intervention (PCI) are commonly employed to restore coronary blood flow and slow disease progression^8–11^. Recent studies have illuminated the roles of ferroptosis and oxidative stress in the pathogenesis of atherosclerosis, which may contribute to the development of ICM. These processes also play a significant role in conditions like diabetes, which can further predispose individuals to DCM^12^. Furthermore, ferroptosis has been increasingly recognized as a key factor in the development of cardiomyopathy, atherosclerosis, and heart failure. Ferroptosis contributes to oxidative stress and inflammation, processes which are central to cardiovascular diseases^13^. Ferroptosis plays a critical role in promoting inflammation and oxidative stress, thereby contributing significantly to the formation of atherosclerotic plaques^14^.

Additionally, emerging evidence suggests that the Bach1/HO-1 pathway regulates oxidative stress and promotes ferroptosis in cardiomyopathies^15^. Thus, oxidative stress likely contributes to the development of cardiomyopathies, arising from an imbalance between the generation of reactive oxygen species (ROS) and the capacity of the oxidative clearance system^16^. Currently, oxidative stress has been recognized as a prominent hallmark in the pathogenesis of heart failure resulting from cardiomyopathies^17,18^. Oxidative stress is strongly linked to ICM.

Oxidative stress is strongly linked to ICM. Vascular smooth muscle cells (VSMCs), in conjunction with fibroblasts and endothelial cells, play a crucial role in the pathogenesis of cardiomyopathies. VSMCs are integral to atherosclerotic plaque formation and contribute to pathological processes such as apoptosis and autophagy^19^. VSMCs also mediate vascular remodeling, a critical aspect of both vascular and myocardial changes^20^. Notably, a study by Ye et al.^21^ demonstrated that VSMCs undergo ferroptosis, which promotes vascular calcification, contributing to the narrowing of coronary arteries and the progression of ICM. Additionally, the proliferation and migration of VSMCs are essential for vascular remodeling and are closely linked to the pathogenesis of atherosclerosis and other vascular diseases^22^. Therefore, smooth muscle cells (SMCs) may contribute to myocardial damage through various mechanisms, including ferroptosis, oxidative stress, proliferation, and migration, leading to the development of different cardiomyopathies. However, research specifically targeting SMCs in cardiomyopathies is limited. The relationship between SMCs and cardiomyopathies, particularly aspects such as cell stemness, transcription factor activity, and intercellular interactions, remains underexplored and warrants further investigation.

This study aims to utilize single-cell RNA sequencing (scRNA-seq) to investigate the spectrum of SMCs involved in cardiomyopathies, particularly ICM, with the goal of providing a comprehensive understanding of their cellular heterogeneity. By identifying specific subpopulations closely associated with ICM onset and progression, this research seeks to integrate single-cell data related to cell cycle, differentiation, stemness, communication, and transcriptional regulation. The identification of potential therapeutic targets within these subpopulations could lead to novel strategies to intervene in the progression of cardiomyopathies and prevent heart failure.

## Materials and methods

### Data collection and processing

The scRNA-seq data for smooth muscle cells in cardiomyopathy were obtained from the GEO database (https://www.ncbi.nlm.nih.gov/geo/) under accession number GSE145154. The numbers of the sample we selected are GSM4307519, GSM4307515, GSM4307516, GSM4307517, GSM4307518, GSM430752, GSM4307520, GSM4307521, GSM4307522, GSM4307523, GSM4307529, GSM4307525, GSM4307526, GSM4307527, GSM4307528, GSM4307530, GSM4307531, GSM4307532, GSM4307533, GSM4307539, GSM4307536, GSM4307538, GSM4307544, GSM4307540, GSM4307541, GSM4307543, GSM4307534, GSM4307545, GSM4307546, GSM4307549, GSM4307550 and GSM4307551. These samples correspond to scRNA-seq data from N-1-Bld, N-1-LVP, N-1-LVN, N-1-RVP, N-1-RVN, DCM-2-Bld, DCM-2-LVP, DCM-2-LVN, DCM-2-RVP, DCM-2-RVN, DCM-3-Bld, DCM-3-LVP, DCM-3-LVN, DCM-3-RVP, DCM-3-RVN, ICM-1-Bld, ICM-1-MIP, ICM-1-MIN, ICM-1-NMIP, ICM-1-NMIN, ICM-2-Bld, ICM-2-LVN, ICM-2-RVN, ICM-3-Bld, ICM-3-LVP, ICM-3-LVN, ICM-3-RVN, RM-36, RM-37, RM-40, RM-42 and RM-44. The 10X Genomics data for each sample were imported into R software (v4.3.3) using the Seurat package (v4.1.1)^23,24^. To maintain data integrity, potential doublet cells were identified and removed using the DoubletFinder program (v2.0.3)^25–27^. Furthermore, we applied a rigorous filtering process to exclude low-quality cells. Specifically, cells were retained only if they met the following quality thresholds: 300 < nFeature < 7500 and 500 < nCount < 100,000. Additionally, cells with mitochondrial gene expression exceeding 20% of the total expression were excluded. Consistent with our study’s criteria, cells with fewer than 500 or more than 6,000 identified genes were excluded as low-quality.

### Dimensionality reduction, clustering, and cell type identification via scRNA-seq

Gene expression in each cell was quantified using the log(x + 1) transformation method, which scales the gene counts to a fraction of 10,000, followed by a natural logarithm transformation^28–30^. To ensure proper normalization, the expression matrix was normalized, and subsequently, the top 2000 highly variable genes (HVG) were identified^31–33^. Principal Component Analysis (PCA)^34,35^ was employed to analyze these genes, and to mitigate potential batch effects across samples, the Harmony method was applied^36–38^. For subsequent dimensionality reduction and clustering, we selected the top 30 principal components (PCs). The results obtained were then visualized on a 2D plot using the UMAP algorithm^39^, facilitating cell type identification^30,40^.

To annotate the resulting cell clusters and identify distinct cell types, we utilized known cell markers from previous literature and the CellMarker database (http://xteam.xbio.top/CellMarker/)^41^. These marker genes were employed to assign cell types to the clusters. Furthermore, for the SMCs identified through this process, additional clustering was performed to classify each SMC subpopulation based on specific marker genes.

### Enrichment analysis and AUCell analysis

Differentially expressed genes (DEGs) for each cell type were identified using the“FindMarkers”function^40,42^ with default parameters, employing the Wilcoxon rank-sum test. DEGs were specifically selected from clusters showing a logFC value greater than 0.25 and expressed in more than 25% of cells within the cluster. To gain further insights into the functional roles of these DEGs within each cell type, enrichment analysis was conducted using the clusterProfiler (v4.6.2) and SCP (v0.4.8) packages. Pathway enrichment analysis utilized Gene Ontology (GO)^43,44^ Biological Process (BP) terms, revealing significantly enriched biological processes and molecular functions associated with the DEGs in each cell type. This analysis provided valuable information about the functional implications of these genes within their respective cellular contexts^45–49^.

Additionally, we integrated a novel method called AUCell to identify active gene sets within our scRNA-seq data^50^. AUCell offers a unique approach to assess the“activity”of specific gene sets within individual cells. Utilizing this method, we obtained outputs that provided valuable information about the activity levels of gene sets across our dataset.

### Slingshot and CytoTRACE analysis

To explore developmental and differentiation variations among distinct subpopulations of SMCs in cardiomyopathy, we utilized the“Slingshot”package (v2.6.0)^51–53^ to delineate the developmental trajectories of each SMC subpopulation. Subsequently, we employed CytoTRACE analysis to rank the differentiation states of all SMC subpopulations^54,55^. The functions“getlineage”and“getCurves”were employed to infer the differentiation trajectories of each SMC subpopulation and evaluate temporal changes in expression levels.

### Construction of pseudotime trajectories for SMC subpopulations

We utilized Monocle (v2.24.0) to construct pseudotime trajectories of SMC subpopulations. Through the analysis of pseudotemporal ordering in scRNA-seq data, Monocle aims to reveal the cellular changes occurring during the redifferentiation process of SMCs.

### Cell–cell communication

To visualize interactions among all cell types, including interactions between SMC subpopulations and endothelial cells, we utilized the CellChat package (v1.6.1) based on scRNA-seq data^56,57^. The CellChatDB.human reference database for ligand-receptor interactions was employed. Intercellular interactions across different cell types were predicted, with a significance threshold set at p-value 0.05^58^.

### Utilization of SCENIC for gene regulatory network reconstruction

To reconstruct gene regulatory networks and identify stable cell states from scRNA-seq data, we utilized the pySCENIC package (v0.12.1) in Python (v3.9.19) with default parameters^59–61^. In this study, we generated AUCell matrices to assess the enrichment of transcription factors (TFs) and the activity of regulatory factors^57^.

### Cell culture

The HA-VSMC cell line was sourced from Procell in Wuhan, China. The cells were cultured in Endothelial Cell Medium (ECM, SCIENCELL) supplemented with 10% fetal bovine serum, penicillin (100 U/mL), and streptomycin (0.1 mg/mL). Cultures were maintained in a standard incubator at 37 °C, 5% CO2, and saturated humidity. Cells in the logarithmic growth phase were harvested for subsequent experiments.

### SiRNA transfection

For the experimental procedure, cells were seeded onto 6-well plates at a density of 2 × 10^5 cells per well. After 24 h, the cells were transfected with siRNAs obtained from GenePharma (Shanghai, China) at a final concentration of 20 µM. Transfection was performed using RNAiMax (Life Technologies, ThermoFisher Distributor; Brendale QLD, Australia) following the manufacturer’s instructions. Cells were collected 24 h post-transfection for further analysis. The sequences of the siRNAs used in the experiment were as follows: siRNA1: UCAACAAGUCAGAACUAAA; siRNA2: UCCAAGAGUACUGUGUCUU; NC siRNA: UUCUCCGAACGUGUCACGU.

### RNA extraction and quantitative real-time PCR

To isolate total RNA, TRIzol reagent from Thermo Fisher Scientific (Waltham, MA, USA) was used following the manufacturer’s instructions. Subsequently, 500 ng of RNA was used for cDNA synthesis using the PrimeScript RT Reagent Kit from TaKaRa (Tokyo, Japan). For quantitative real-time PCR, the SYBR® Premix Ex Taq™ from TaKaRa was utilized. PCR was conducted on the ABI V7 instrument from ABI (Indianapolis, IN, USA). The specific primers used for amplification were designed for the S100A4 gene as follows: Forward primer: 5’-CGAGGTGGACTTCCAAGAGT-3’ and Reverse primer: 5’-TCATTTCTTCCTGGGCTGCT-3’. In addition, the housekeeping gene we used was GAPDH, and its primer sequence was as follows: GAPDH-homo-qF: GGACTCATGACCACAGTCCA; GAPDH-homo-qR: TCAGCTCAGGGATGACCTTG.

### Analysis of western blotting

Lysis buffer (1 M Tris–HCL, pH 6.8, 10% SDS, and 80% glycerin) was used to remove all of the cellular protein. Following the manufacturer’s instructions, we utilized a bicinchoninic acid (BCA) test to find out how much protein was in the sample. In short, a 10% SDS-PAGE gel was used to separate the 30 μg of total protein, and then the proteins were transferred to polyvinylidene fluoride (PVDF) membranes (Millipore; Burlington, MA, USA) using electrophoresis. Next, the membranes were washed three times with Tris buffered saline plus Tween (TBST) and then completely blocked with 5% skim milk. We incubated the blocked PVDF membranes with primary antibodies overnight at 4 °C. Secondary antibodies were used for two hours after the original antibodies had been incubated. We used an enhanced chemiluminescence kit (ECL; ThermoFisher Scientific, Waltham, MA, USA) to check if we could see the protein bands.

### Cell viability assay

Cell viability was evaluated using the Cell Counting Kit-8 (CCK-8) from DOJINDO (Kumamoto, Japan). Cells were seeded at 1 × 10^3 cells per well in 96-well plates and cultured overnight. A 100 µL detection reagent was added to each well and incubated for 1 h. Absorbance at 450 nm was measured daily over 4 days. Growth curves were plotted by correlating OD450 values with time to assess cell viability.

### Clone formation assay

Logarithmically growing cell suspensions were diluted and plated into 6-well plates at a density of 1 × 10^3 cells per well. The cells were cultured for 10 days with regular monitoring. Upon visible clone formation, the culture medium was removed, and cells were washed twice with ice-cold PBS. Subsequently, cells were fixed in 4% paraformaldehyde for 20 min, followed by staining with 0.1% crystal violet for 10 min. Colonies were then counted using the Gel imaging analysis system (Syngene, GBOX-F3EE; Bangalore, India).

### Edu analysis

Logarithmically growing cell suspensions were prepared, diluted, and plated into 6-well plates at a density of 1 × 10^3 cells per well. An EdU assay was conducted according to the manufacturer’s instructions (RiboBio, China). Treated cells were visualized using fluorescence microscopy, and quantification was performed by counting at least 6 random fields.

### Wound healing assay

Cells were seeded at a density of 2 × 10^5 cells per well in 6-well plates and cultured overnight. A scratch was generated in the cell monolayer using a 10 µL pipette tip held perpendicular to the culture plate. The cells were then washed three times with PBS to remove any detached cells, and fresh serum-free medium was added to the plates. Subsequently, the cells were incubated at 37 °C in a 5% CO2 chamber. Bright-field microscopy was employed to capture images of the scratch area at 0 h and 48 h.

### Transwell migration and invasion assay

A Transwell assay was performed to evaluate cell migration and invasion. Cells were seeded in 24-well Transwell insert chambers (BD Biosciences, USA) with serum-free medium. For the invasion assay, the insert chambers were pre-coated with 2% Matrigel. The lower chamber medium was supplemented with 20% FBS, acting as a chemo-attractant. After a 2-day incubation period, non-migrated cells on the upper side of the membrane were gently removed. Cells that had migrated or invaded through the membrane were fixed and stained with crystal violet. Finally, the migrated or invaded cells were observed and photographed under a microscope.

### Flow cytometry for apoptosis analysis

To evaluate cellular apoptosis, the Annexin V-FITC/PI apoptosis detection kit from Sigma-Aldrich, Germany, was employed. Cells were incubated and washed with cold PBS, followed by suspension in a binding buffer containing Annexin V-FITC and PI stains. After incubation in the dark, the stained cells were analyzed using flow cytometry. Flow cytometry data was processed using FlowJo (v10.0.7) software to quantify the extent of apoptosis within the cell population.

### Statistical analysis

Statistical analysis was performed using R software (v4.3.3) and Python software (v3.9.19). Wilcoxon’s test and Pearson correlation coefficient were employed to assess the significance of differences between different groups. Significance levels were indicated as follows: *P < 0.05, **P < 0.01, ***P < 0.001, ****P < 0.0001. The term“ns”was used to denote non-significant differences between groups. These statistical tests and significance indicators were utilized to evaluate the statistical significance of the findings and provide confidence in the results.

## Results

### Overview of the study

**Fig. 1:**
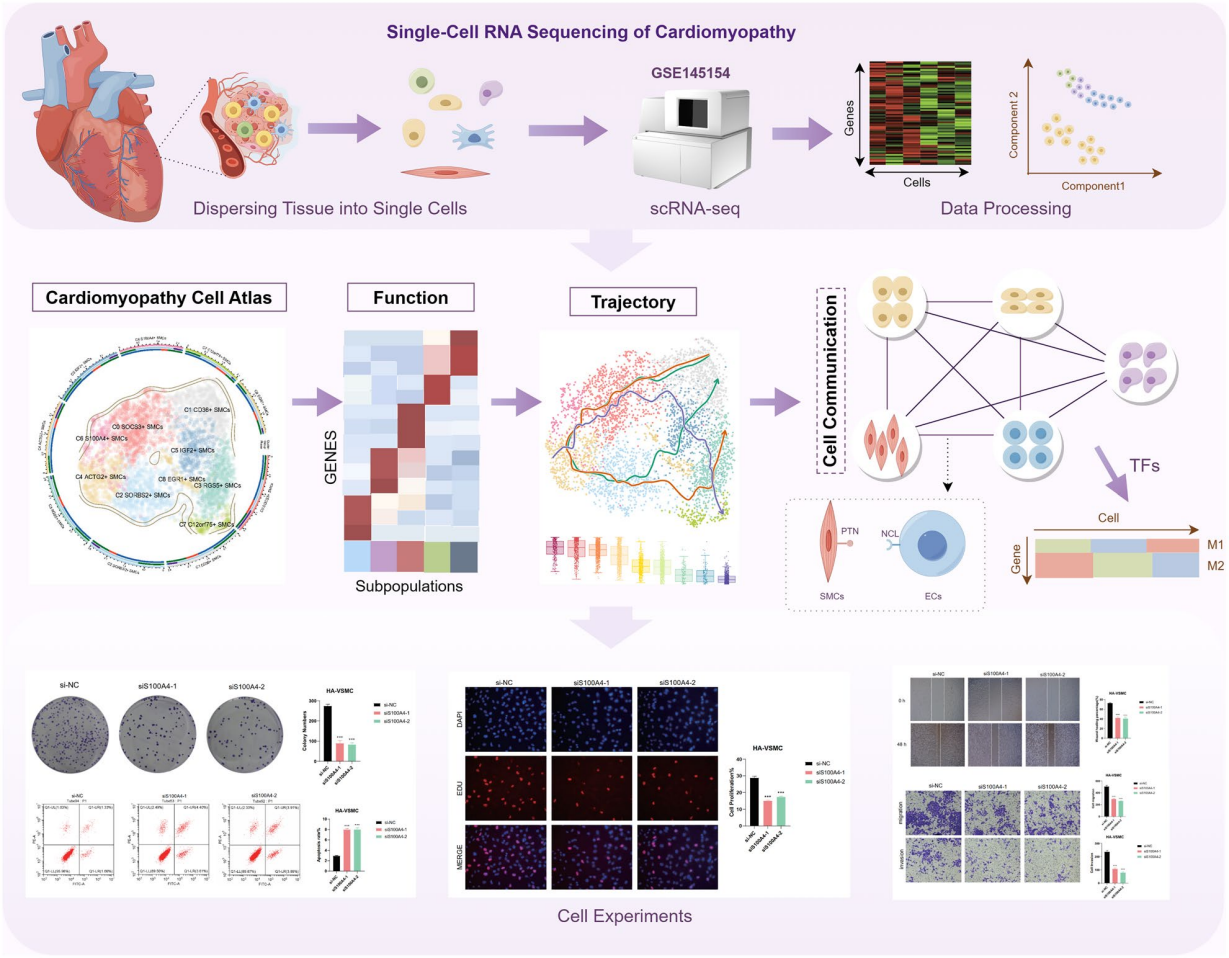
Principles of research.

Cardiomyopathy is strongly associated with ferroptosis. The scRNA-seq of 4,184 SMCs found that C6 S100A4 + SMCs were critical, exhibiting high expression of ferroptosis, oxidative stress, and inflammation, which we demonstrated through in vitro experiments. In addition, pathways such as PTN-NCL may provide new treatments **(**Fig. 1**)**.

### Identification and characterization of distinct smooth muscle cell subpopulations in cardiomyopathy

**Fig. 2:**
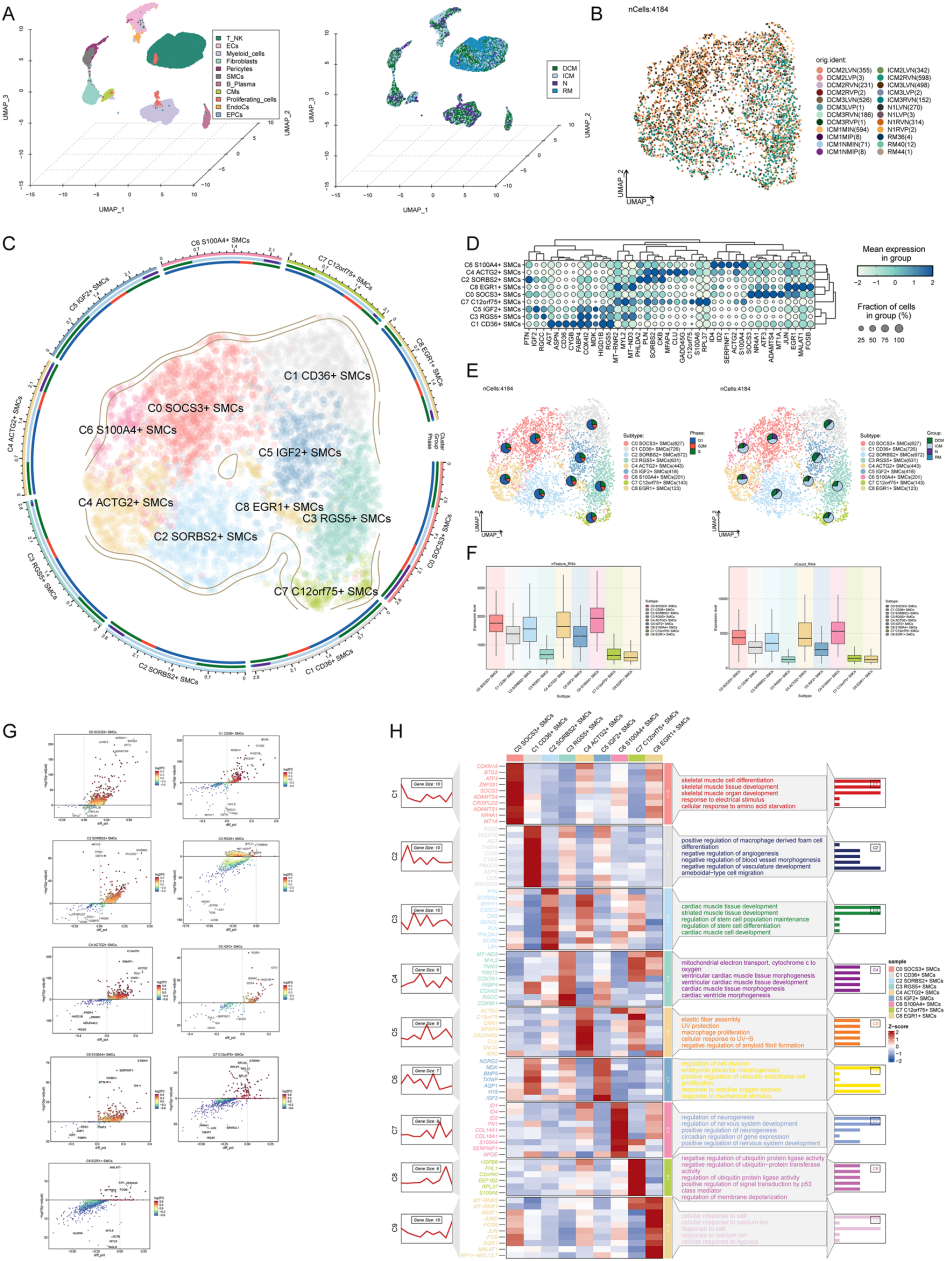
Visualization of smooth muscle cell subpopulations in cardiomyopathy (**A**) The spatial distribution of all cells in the selected cardiomyopathy samples (left) and their distribution across different groups (right) were depicted in a 3D UMAP plot. (**B**) The UMAP plot illustrated the specific distribution of 24 samples selected from GSE145154. (**C**) Smooth muscle cells were categorized into nine subpopulations based on differential expression of marker genes. The UMAP plot displayed their distributions and provided information about the proportions of different groups and cell cycles within each subpopulation. (**D**) Average expression levels of differentially expressed genes in the nine smooth muscle cell subpopulations were presented in a bubble plot. (**E**) Pie charts directly showed the proportions of cell cycles and different groups within each subpopulation on the UMAP plot. (**F**) Box plots illustrated the nFeature_RNA and nCount_RNA of the nine smooth muscle cell subpopulations. (**G**) Volcano plots depicted the top 5 upregulated and top 5 downregulated genes in the nine smooth muscle cell subpopulations. (**H**) Highly expressed differential genes in the nine smooth muscle cell subpopulations were subjected to GO-BP enrichment analysis.

Following batch correction, The sample sources of high-quality cells are shown in Figure S1A. we categorized 179,927 high-quality cells into various cell types, including T cells, NK cells, endothelial cells (ECs), myeloid cells, fibroblasts, pericytes, SMCs, B cells, plasma cells, cardiomyocytes (CMs), proliferating cells, EndoCs, and endothelial progenitor cells (EPCs). These samples were stratified into four groups based on cardiomyopathy types: DCM, ICM, RM, and normal (N). Utilizing UMAP plots, we observed that SMCs were predominantly associated with ICM and DCM cases (Fig. 2A). All high-quality cells were in different cell cycle stages(Figure S1B). Further batch correction enabled the identification of 4,184 SMCs from 24 samples, which were segmented into nine distinct subpopulations characterized by specific marker genes: C0 SOCS3+ SMCs, C1 CD36+ SMCs, C2 SORBS2+ SMCs, C3 RGS5+ SMCs, C4 ACTG2+ SMCs, C5 IGF2+ SMCs, C6 S100A4+ SMCs, C7 C12orf75+ SMCs, and C8 EGR1+ SMCs (Figs. 2B and C). All subpopulations exhibited differences in cell cycle distribution, with the majority of cells being in the G1 phase (Figure S1C). Each subpopulation exhibited unique highly expressed genes alongside their defining markers. For example, C6 S100A4+ SMCs demonstrated elevated expression of ID4, ID2, SERPINF1, and ACTG2, while C4 ACTG2+ SMCs displayed heightened levels of PLN, SORBS2, CKB, MFAP4, CLU, and GADD45G (Fig. 2D). S100A4 was expressed at the highest level in the C6 subpopulation, and was therefore selected as the marker gene (Figures S1D-E). Analysis of cell cycle phases within these SMC subpopulations revealed a predominant presence in the G1 phase, with C6 S100A4+ SMCs showing the highest proportion in G1 and the lowest in G2M phases. Notably, C6 S100A4+ SMCs were predominantly derived from the ICM group, with other subpopulations also exhibiting significant contributions from ICM. However, C0 SOCS3+ SMCs showed nearly equal proportions from DCM, ICM, and N groups (Fig. 2E). Additionally, C6 S100A4+ SMCs exhibited the highest nFeature_RNA and nCount_RNA values, followed by C0 SOCS3+ SMCs, whereas C8 EGR1+ SMCs displayed the lowest values **(**Fig. 2F**).** Volcano plots were utilized to visualize top upregulated and downregulated genes in each SMC subpopulation. For instance, in C6 S100A4+ SMCs, top upregulated genes included S100A4, SERPINF1, INHBA, SFTA1P, and ID4, while top downregulated genes encompassed FABP4, IGF2, AQP1, TESC, and TIMP3. Similarly, other subpopulations exhibited distinct gene expression profiles **(**Fig. 2G**)**. To gain insights into biological processes associated with each subpopulation in cardiomyopathy progression, we conducted GO-BP enrichment analysis. Genes with high Z-score differentials in C6 S100A4 + SMCs, such as ID1, ID4, ID2, FN1, COL14A1, COL18A1, S100A4, SERPINF1, and APOE, were significantly enriched in processes such as regulation of neurogenesis, regulation of nervous system development, positive regulation of neurogenesis, circadian regulation of gene expression, and positive regulation of nervous system development **(**Fig. 2H**)**.

### Differences in stemness and oxidative phosphorylation among SMC subpopulations, with a focus on C6 S100A4 + SMCs

**Fig. 3:**
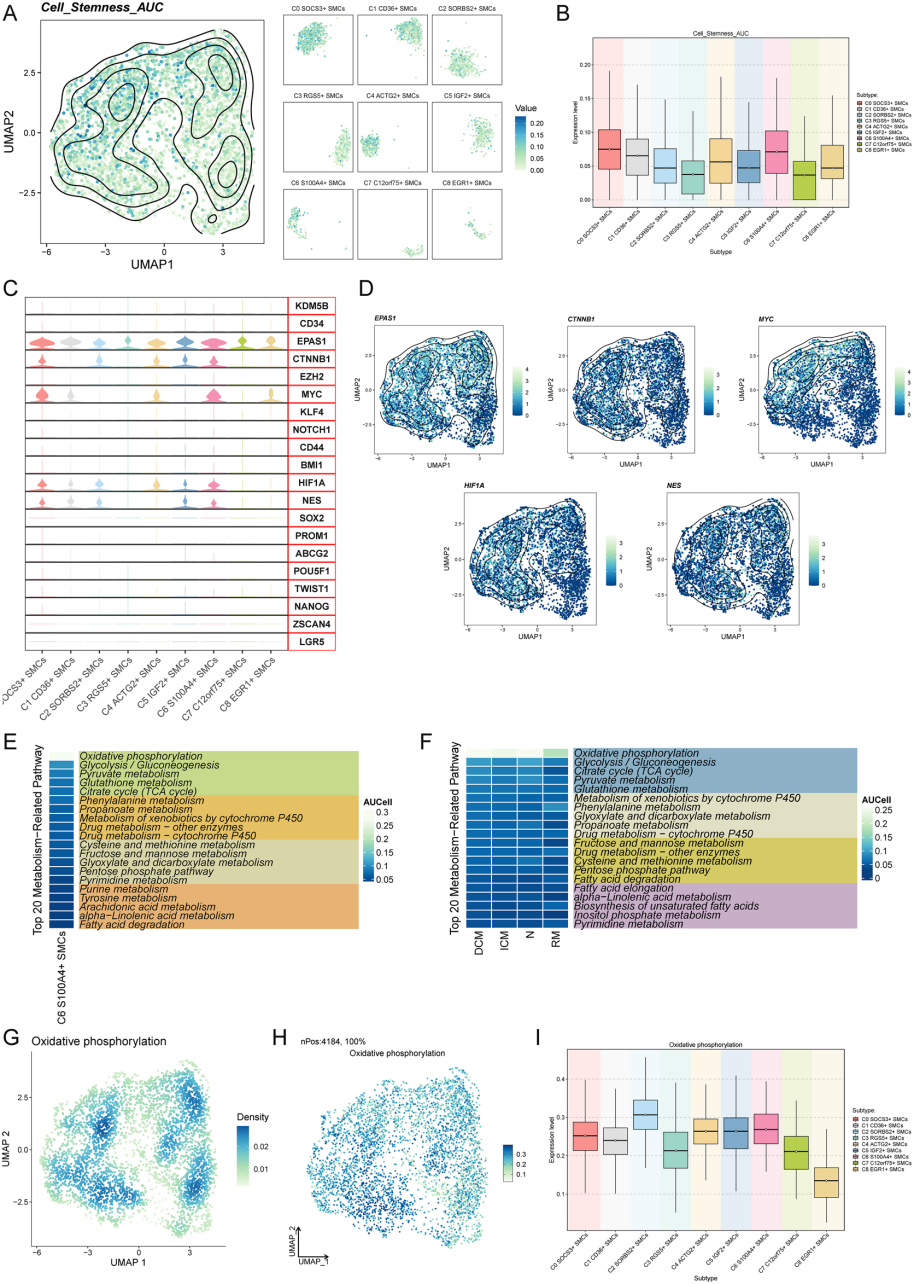
Stemness and metabolic activity of smooth muscle cell subpopulations (**A**) The distribution of stemness AUC values across the nine smooth muscle cell subpopulations was visualized using UMAP plots and faceted plots. (**B**) Box plots provided a clear comparison of stemness AUC values among the nine smooth muscle cell subpopulations. (**C**) Violin plots illustrated the differential expression of stemness genes across the nine smooth muscle cell subpopulations. (**D**) UMAP plots displayed the expression and density distribution of five highly expressed stemness genes (EPAS1, CTNNB1, MYC, HIF1A, NES) across all smooth muscle cell subpopulations. (**E**) Heatmaps depicted the AUC values of the top 20 metabolic pathways in C6 S100A4+ SMCs. (**F**) Heatmaps presented the AUC values of the top 20 metabolic pathways across the four groups. (**G**) UMAP plots showed the density distribution of the oxidative phosphorylation pathway. (**H**) UMAP plots displayed the expression intensity distribution of the oxidative phosphorylation pathway. (**I**) Box plots visually represented differences in the expression levels of the oxidative phosphorylation metabolic pathway among the nine smooth muscle cell subpopulations.

To explore the proliferative characteristics of SMC subpopulations, we conducted a stemness scoring analysis. Initially, we visualized the distribution of stemness AUC values across the nine SMC subpopulations using UMAP and facet plots **(**Fig. 3A**)**. Box plots were employed for a comprehensive comparison, revealing that C6 S100A4+ SMCs and C0 SOCS3+ SMCs displayed the highest stemness AUC values (Fig. 3B).

Furthermore, we identified several genes with elevated stemness expression in these SMC subpopulations, including EPAS1, CTNNB1, MYC, HIF1A, and NES **(**Fig. 3C**)**. UMAP plots were then used to illustrate the distribution and density of AUC values for these five stemness genes within the subpopulations **(**Fig. 3D**)**. Notably, strong cellular stemness often correlates with heightened metabolic activity, indicative of high proliferative capacity, active cell growth, and division. Therefore, we performed AUCell scoring on the metabolic pathways of C6 S100A4 + SMCs and represented the top 20 pathways in a heatmap. Our analysis underscored oxidative phosphorylation as the most active metabolic pathway in C6 S100A4+ SMCs **(**Fig. 3E**)**.

To further explore the role of oxidative phosphorylation across different groups, we conducted AUCell scoring of this pathway. The results indicated that the N group exhibited the highest scores, followed by the DCM and ICM groups **(**Fig. 3F**)**. UMAP plots were utilized to visualize the density and expression levels of oxidative phosphorylation within the SMC subpopulations **(**Figs. 3G and H**).** Finally, comparing the rankings of oxidative phosphorylation scores among all SMC subpopulations revealed that C2 SORBS2+ SMCs had the highest scores, followed by C6 S100A4+ SMCs, while C8 EGR1+ SMCs exhibited the lowest scores **(**Fig. 3I**)**.

### Differences in ferroptosis and oxidative stress among SMC subpopulations

To investigate the potential association between SMC subpopulations and the development of cardiomyopathy, particularly ICM, we examined the roles of ferroptosis and oxidative stress, both of which are implicated in coronary atherosclerosis and subsequent cardiomyopathy. We conducted scoring for ferroptosis and oxidative stress-related pathways across all SMC subpopulations.

**Fig. 4:**
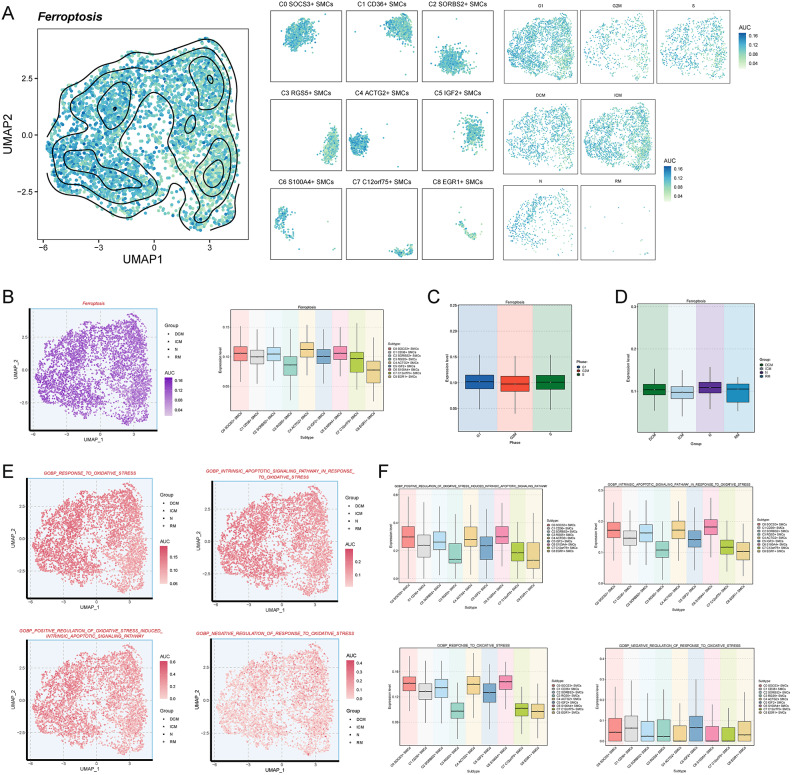
Differences in ferroptosis and oxidative stress scores across all smooth muscle cell subpopulations (**A**) UMAP plots were used to visualize the distribution of ferroptosis AUC values. Faceted plots provided detailed insights into the distribution differences of ferroptosis AUC values within each subpopulation, across different groups, and during different cell cycles. (**B**) Detailed variations in ferroptosis scores were illustrated, highlighting specific groups within each subpopulation. Comparisons of ferroptosis scores among the nine smooth muscle cell subpopulations were also presented. (**C**) Variations in ferroptosis scores across different cell cycles were displayed. (**D**) Variations in ferroptosis scores across different groups were illustrated. (**E**) UMAP plots depicted distribution differences of four oxidative stress-related scores. (**F**) Box plots visually compared the differences in the four oxidative stress-related scores across the different smooth muscle cell subpopulations.

Initially, we visualized the distribution of ferroptosis AUC values across the nine SMC subpopulations using UMAP and facet plots, segmented by different cell cycle phases and groups **(**Fig. 4A**)**. Overlaying these values onto a unified UMAP plot with different shapes representing distinct groups, we observed that ferroptosis AUC values were notably elevated in C4 ACTG2+ SMCs, C6 S100A4+ SMCs, and C0 SOCS3+ SMCs **(**Fig. 4B**)**. Differences in ferroptosis scores across cell cycle phases were minimal, with slightly higher scores observed during G1 and S phases compared to the G2M phase **(**Fig. 4C**)**. Similarly, variations in scores across the four groups (N, DCM, ICM, RM) were minor, with slightly higher scores in the N and RM groups **(**Fig. 4D**)**.

Next, we assessed four gene sets related to oxidative stress pathways across all SMC subpopulations. These pathways included response to oxidative stress, intrinsic apoptotic signaling pathway in response to oxidative stress, positive regulation of oxidative stress-induced intrinsic apoptotic signaling pathway, and negative regulation of response to oxidative stress. Using UMAP plots, we depicted the distribution and strength of pathway scores across different subpopulations and groups, where shapes represented different groups **(**Fig. 4E**)**. Box plots revealed that C6 S100A4+ SMCs exhibited the highest scores in response to oxidative stress, intrinsic apoptotic signaling pathway in response to oxidative stress, and positive regulation of oxidative stress-induced intrinsic apoptotic signaling pathway **(**Fig. 4F**)**. Interestingly, C6 S100A4 + SMCs, along with C4 ACTG2+ SMCs and C7 C12orf75+ SMCs, showed the lowest scores in negative regulation of response to oxidative stress.

### Slingshot analysis reveals developmental and differentiation characteristics of SMC subpopulations

**Fig. 5:**
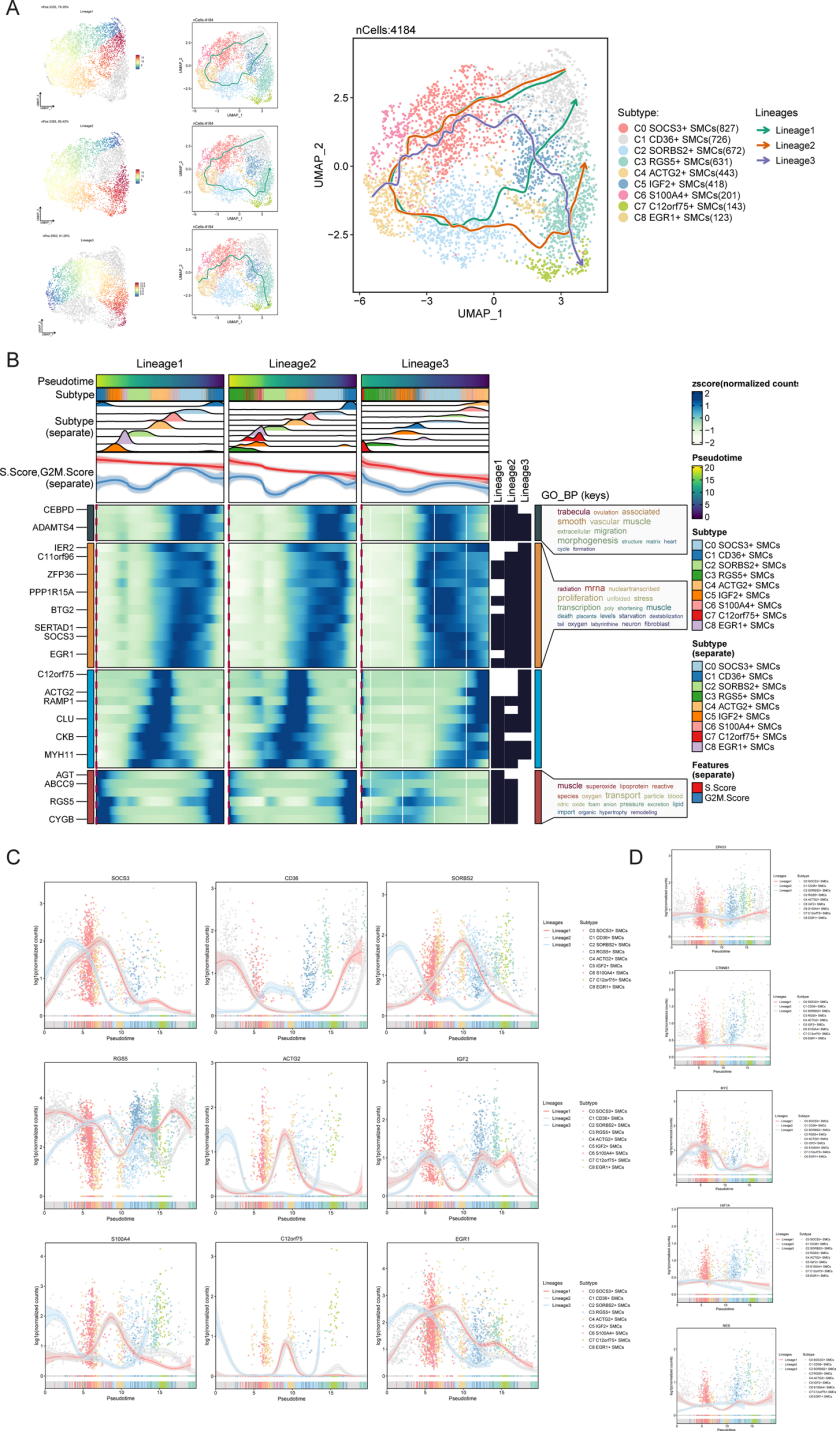
Slingshot analysis of smooth muscle cell subpopulations (**A**) Three fitted differentiation trajectories of smooth muscle cell subpopulations were depicted. The temporal order and distribution of these trajectories were further validated using UMAP plots. The trajectories were as follows: Lineage 1: C1 → C0 → C6 → C4 → C2 → C8 → C5 → C1; Lineage 2: C1 → C0 → C6 → C4 → C2 → C3; Lineage 3: C4 → C6 → C0 → C1 → C5 → C3 → C7. (**B**) Heatmaps illustrated the differential expression of genes along the three trajectories, accompanied by their GO-BP enrichment analysis. (**C**) The differential expression of marker genes in the nine smooth muscle cell subpopulations was shown as they progressed through the three trajectories. (**D**) The differential expression of the five aforementioned stemness genes (EPAS1, CTNNB1, MYC, HIF1A, NES) was presented as they evolved along the three trajectories.

To gain insights into the developmental stages and differentiation levels of SMC subpopulations, we employed Slingshot analysis to model developmental trajectories, identifying three distinct SMC lineage trajectories: Lineage 1, Lineage 2, and Lineage 3. Lineage 1: C1 CD36+ SMCs → C0 SOCS3+ SMCs → C6 S100A4+ SMCs → C4 ACTG2+ SMCs → C2 SORBS2+ SMCs → C8 EGR1+ SMCs → C5 IGF2+ SMCs → C1 CD36+ SMCs; Lineage 2: C1 CD36+ SMCs → C0 SOCS3+ SMCs → C6 S100A4+ SMCs → C4 ACTG2+ SMCs → C2 SORBS2+ SMCs → C3 RGS5+ SMCs; Lineage 3: C4 ACTG2+ SMCs → C6 S100A4+ SMCs → C0 SOCS3+ SMCs → C1 CD36+ SMCs → C5 IGF2+ SMCs → C3 RGS5+ SMCs → C7 C12orf75+ SMCs. In our analysis, we utilized UMAP plots to visualize the sequential expression of SMC lineage trajectories across all subpopulations, aligning them with a temporal order observed in another UMAP plot **(**Fig. 5A**)**. This allowed us to observe how different subpopulations progressed along distinct differentiation paths.

Next, we investigated differential gene expression along the three identified differentiation trajectories, focusing particularly on the characteristics of C6 S100A4+ SMCs within these trajectories. Our exploration revealed several genes closely associated with C6 S100A4+ SMCs, including IER2, C11orf96, ZFP36, PPP1R15A, BTG2, SERTAD1, SOCS3, and EGR1.

Furthermore, we conducted GO-BP enrichment analysis on the differentially expressed genes related to SMC subpopulations **(**Fig. 5B**)**. This analysis highlighted enrichment in key biological processes such as radiation response, mRNA processing, nuclear transcription, proliferation, stress response, transcription regulation, muscle development, cell death, and mRNA destabilization, underscoring their relevance in SMC differentiation and function. Additionally, we explored the expression timing of nine selected genes across the three lineages. Notably, S100A4 exhibited early expression in Lineage 3 and mid-stage expression in Lineages 1 and 2 **(**Fig. 5C**)**.

Here are the expression patterns observed: EPAS1: Highly expressed early in Lineage 1 and in the mid-stage of Lineage 3. CTNNB1: Shows a similar expression pattern across the three lineages, with slightly higher mid-stage expression. MYC: Primarily expressed early in all three lineages, with significantly higher expression compared to other stages. HIF1A: Predominantly expressed early in Lineage 3, with a noticeable downregulation later; slightly upregulated mid-stage in Lineages 1 and 2 before downregulation. NES: Upregulated mid-stage in Lineage 3 before significant downregulation; early and late upregulation in Lineages 1 and 2 with mid-stage downregulation **(**Fig. 5D**)**.

### Pseudotime analysis of SMC subpopulations

**Fig. 6:**
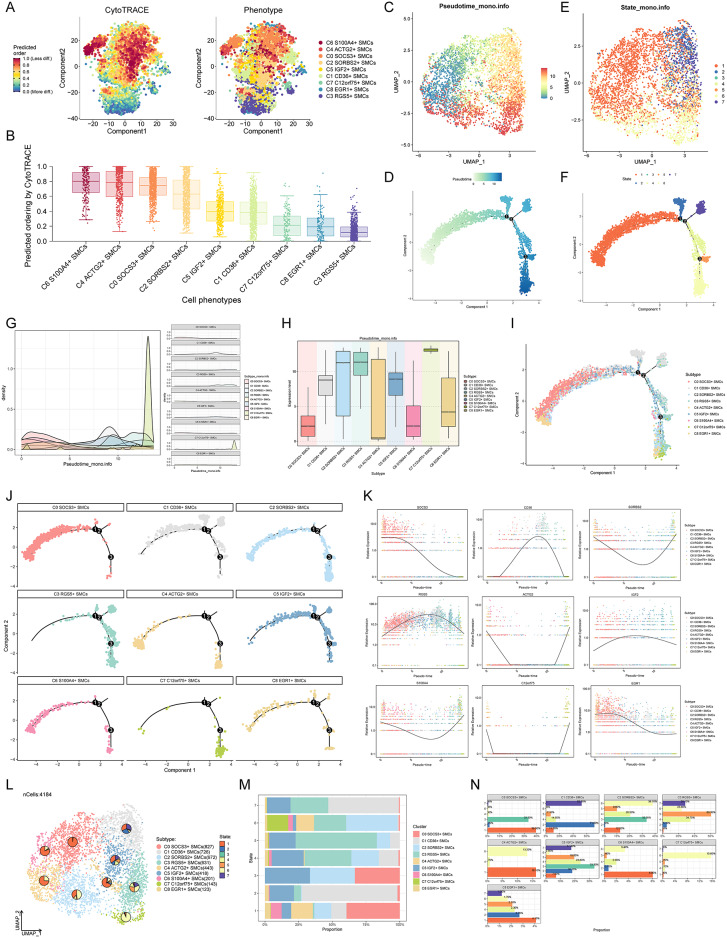
CytoTRACE analysis and pseudotime trajectory validation of smooth muscle cell subpopulations (**A**) UMAP plots displayed the results of CytoTRACE analysis conducted on smooth muscle cell subpopulations. (**B**) Based on the CytoTRACE results, the differentiation levels of the nine smooth muscle cell subpopulations were visualized through box plots. (**C**) UMAP plots showcased the distribution of pseudotime. (**D**) A two-dimensional plot further elucidated the pseudotime trajectory, progressing from left to right with two branches in the middle phase and one branch in the late phase. (**E**) Due to branching in the pseudotime trajectory, the entire pseudotime was divided into seven stages (State 1–7), and their distribution across all smooth muscle cell subpopulations was displayed using UMAP plots. (**F**) A dedicated two-dimensional plot specifically depicted the distribution of State 1–7 along the established pseudotime trajectory. (**G**) Ridge plots displayed the distribution of the nine smooth muscle cell subpopulations on the pseudotime trajectory. (**H**) Box plots provided a more intuitive quantification of the expression levels of the nine smooth muscle cell subpopulations on the pseudotime trajectory. (**I**) A two-dimensional plot visualized the specific distribution of the nine smooth muscle cell subpopulations on the established pseudotime trajectory. (**J**) The distribution characteristics of each smooth muscle cell subpopulation on the trajectory were individually displayed in sequence. (**K**) Visualization of the expression patterns of marker genes for each smooth muscle cell subpopulation over time on the pseudotime trajectory. (**L**) Pie charts on UMAP plots revealed the proportions of different states within each smooth muscle cell subpopulation. (**M**) Bar graphs depicted the proportions of different smooth muscle cell subpopulations within each state. (**N**) Bar graphs showed the percentage of each state occupied by each smooth muscle cell subpopulation.

To validate the findings from Slingshot analysis, we utilized CytoTRACE to assess differentiation levels among SMC subpopulations. CytoTRACE results confirmed that C6 S100A4 + SMCs exhibited the lowest differentiation level, while C3 RGS5 + SMCs showed the highest **(**Figs. 6A and B**)**.

Subsequently, we constructed a pseudotime trajectory using Monocle and visualized it via a UMAP plot, illustrating the temporal progression of each SMC subpopulation **(**Fig. 6C**)**. The detailed trajectory **(**Fig. 6D**)** revealed three branches as time unfolds. Based on these branches, we segmented the trajectory into seven states, shown in Fig. 6F, and mapped these states onto the UMAP plot depicting SMC subpopulations **(**Fig. 6E**)**.

To examine the positional differences of all SMC subpopulations along the pseudotime trajectory, we generated a ridge plot **(**Fig. 6G**)**. This analysis indicated that C0 SOCS3+ SMCs, C4 ACTG2+ SMCs, C6 S100A4+ SMCs, and C8 EGR1+ SMCs primarily occupied early pseudotime stages, while C1 CD36+ SMCs, C2 SORBS2+ SMCs, and C3 RGS5+ SMCs were predominantly in the middle to late stages, and C7 C12orf75+ SMCs were mainly in the latest stage. These findings were further validated by box plots **(**Fig. 6H**)** and illustrated the distribution across pseudotime **(**Fig. 6I**)**, as well as individually showcasing each SMC subpopulation **(**Fig. 6J**)**.

We further examined the expression patterns of nine specified genes across pseudotime in SMC subpopulations **(**Fig. 6K**)**. These genes exhibited diverse expression dynamics, with some upregulated at pseudotime extremes and downregulated in the middle. Additionally, we mapped the seven defined states onto the UMAP plot, using pie charts to display the distribution of each SMC subpopulation within these states **(**Fig. 6L**)**, further substantiated by proportions shown in a bar graph **(**Fig. 6M**)**. In addition, we used a bar chart to show the proportion of state1-7 in each subpopulation (The sum of the proportions of each state in each subpopulation is 1) **(**Fig. 6N**).**

### Interaction between C6 S100A4 + SMCs and ECs via the PTN-NCL signaling pathway

**Fig. 7:**
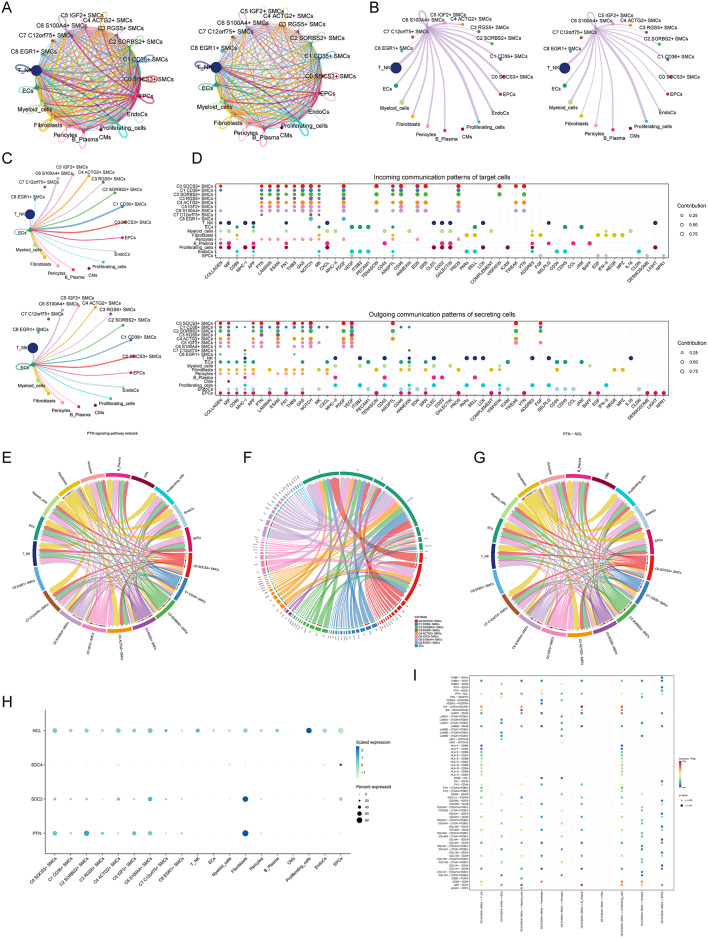
Cell communication landscape in cardiomyopathy (**A**) The left plot depicted the abundance of interactions among all cells in cardiomyopathy, represented as a circle diagram. The right plot visualized the intensity of these interactions among all cells, also shown as a circle diagram. (**B**) In the left plot, the quantity of interactions was shown when C6 S100A4+ SMCs acted as signal emitters to all other cells. The right plot showcased the strength of these interactions when C6 S100A4+ SMCs acted as signal emitters to all other cells. (**C**) The upper plot displayed the quantity of interactions when ECs acted as signal receivers from all other cells. The lower plot presented the strength of interactions when ECs acted as signal receivers from all other cells. (**D**) Bubble charts depicted the expression levels of distinct proteins when all cells acted as signal receivers (top) and when all cells functioned as signal emitters (bottom). (**E**) Chord diagrams illustrated the patterns of interaction among all cells within the PTN signaling pathway network. (**F**) Chord diagrams demonstrated the differential expression of receptor proteins when ECs acted as signal receivers from all smooth muscle cell subpopulations. (**G**) The interaction patterns among all cells within the PTN-NCL receptor-based cell interaction pathway. (**H**) Bubble charts depicted the stable expression levels of PTN protein in C6 S100A4+ SMCs and NCL protein in ECs, respectively. (**I**) Bubble charts presented the expression levels of various receptor proteins when C6 S100A4+ SMCs interacted with other cells (excluding other smooth muscle cell subpopulations).

To explore the potential interaction between C6 S100A4+ SMCs and ECs, we employed CellChat for intercellular interaction analysis. Figure 7A presents a comprehensive overview of interaction quantity and strength among all cell types in myocardial disease samples. Our focus was on C6 S100A4+ SMCs as signal senders interacting with other cells. Notably, C6 S100A4+ SMCs showed robust interactions with T cells, NK cells, myeloid cells, and proliferating cells **(**Fig. 7B**)**.

Subsequently, we examined ECs as signal receivers. ECs received signals from various cell types except CMs and C7 C12orf75+ SMCs. Importantly, ECs received prominent and intense signals from C6 S100A4+ SMCs, C0 SOCS3+ SMCs, and fibroblasts **(**Fig. 7C**)**, underscoring a significant relationship between C6 S100A4+ SMCs and ECs. To delve deeper into the interaction between C6 S100A4+ SMCs and ECs, we investigated protein pathways involved. A bubble plot **(**Fig. 7D**)** visualized protein expression differences when cells acted as signal senders and receivers. Notable proteins highly expressed in ECs included CD99, APP, MIK, VEGF, ITGB2, PECAM1, ANGPTL, SELL, CDH5, and JAM, whereas C6 S100A4+ SMCs exhibited high expression of COLLAGEN, MIF, APP, PTN, ESAM, THBS, GAS, NOTCH, PDGF, and VEGF.

Further analysis focused on the PTN signaling pathway network **(**Figs. 7E and F**)**, revealing a specific interaction between PTN from C6 S100A4+ SMCs and NCL protein on ECs, confirmed through visualizations. A chord diagram **(**Fig. 7G**)** depicted the PTN-NCL signaling pathway, illustrating how C6 S100A4+ SMCs interact with ECs. Additionally, bubble plots **(**Fig. 7H and I**)** verified PTN expression in C6 S100A4 + SMCs and NCL presence in ECs, highlighting the PTN-NCL pathway among various signaling pathways through which these cells interact.

### Gene regulatory network analysis of SMC subpopulations

**Fig. 8:**
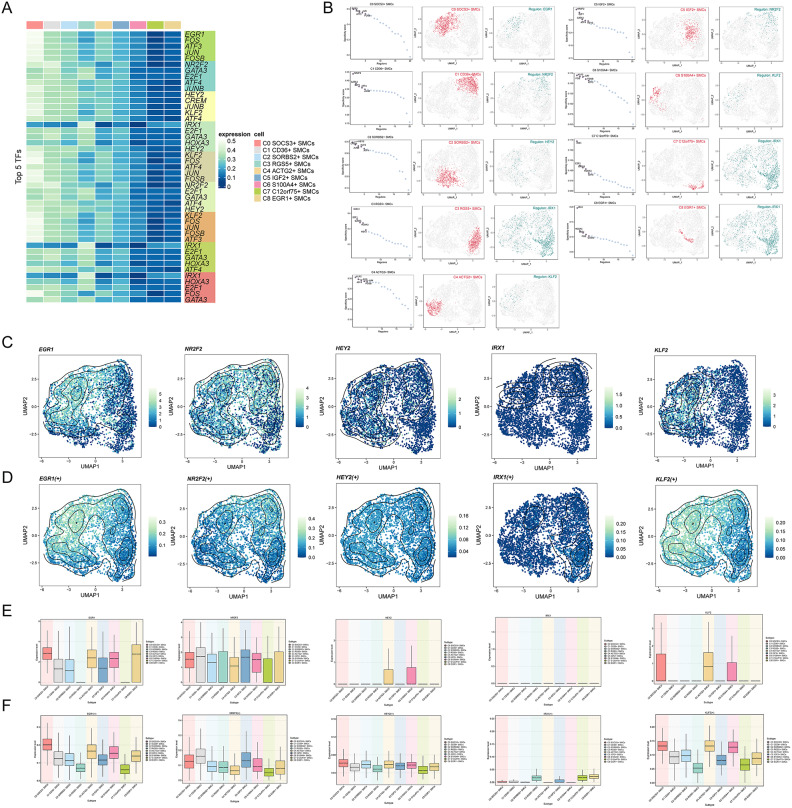
Gene regulatory network analysis of smooth muscle cell subpopulations in cardiomyopathy (**A**) Heatmaps displayed the differential expression of the top 5 TFs in the nine smooth muscle cell subpopulations. (**B**) Regulators in smooth muscle cell subpopulations associated with cardiomyopathy were ranked based on the Regulator Specificity Score (RSS) (left). Each smooth muscle cell subpopulation was highlighted with a red dot in the UMAP plots (middle). The distribution of the highest regulatory subunits, mapped on UMAP plots based on the binary RAS (normalized using Z-score across all samples, with a threshold of 2.5 set to convert to 0 or 1), was highlighted with green dots. (**C**) UMAP plots illustrated the expression levels of EGR1, NR2F2, HEY2, IRX1, and KLF2 in the smooth muscle cell subpopulations. (**D**) UMAP plots depicted the distribution of AUC values for EGR1, NR2F2, HEY2, IRX1, and KLF2 in the smooth muscle cell subpopulations. (**E**) Box plots quantified the differential expression levels of the top 5 highly active regulatory subunits in the nine smooth muscle cell subpopulations. (**F**) Box plots visually compared the AUC values of the aforementioned 5 regulators in the nine smooth muscle cell subpopulations. We used R software (v.4.3.3) (https://cran.r-project.org/) to visualize the results calculated by the pySCENIC module (v0.12.1) based on Python (v3.9.19) (https://www.python.org/). Finally, we integrated all the images in Adobe Illustrator (2024) (https://www.adobe.com/products/illustrator.html).

To identify core TFs associated with myocardial disease in SMC subpopulations, we utilized PySCENIC for analysis. Initially, we determined the top5 TFs for each SMC subpopulation **(**Fig. 8A**)**. For C6 S100A4+ SMCs, these TFs were identified as KLF2, FOS, JUN, FOSB, and ATF3. We then ranked regulatory factors based on their regulon specificity score (RSS) and highlighted specific subpopulations with red dots on the UMAP plot. Additionally, we illustrated the distribution of binary regulon activity scores (RAS) of the top regulons on the UMAP plot with green dots, consistent with the heatmap **(**Fig. 8B**)**.

In summary, the top 5 active TFs identified were EGR1, NR2F2, HEY2, IRX1, and KLF2. Notably, KLF2 emerged as the most active TF in both C4 ACTG2+ SMCs and C6 S100A4+ SMCs. NR2F2 showed prominence as the top TF in both C1 CD36+ SMCs and C5 IGF2 + SMCs. IRX1 was notably active in C3 RGS5+ SMCs, C7 C12orf75+ SMCs, and C8 EGR1+ SMCs.

We displayed the expression levels and density distribution of these 5 TFs sequentially on the UMAP plots **(**Fig. 8C**)** alongside their distribution of AUC values **(**Fig. 8D**)**. To compare expression intensity differences among these TFs in each subpopulation, we used boxplots. EGR1 exhibited higher expression in C0 SOCS3+ SMCs, C8 EGR1+ SMCs, C4 ACTG2+ SMCs, and C6 S100A4+ SMCs. NR2F2 showed higher expression in C1 CD36+ SMCs, C5 IGF2+ SMCs, C0 SOCS3+ SMCs, and C6 S100A4+ SMCs. HEY2 was predominantly expressed in C4 ACTG2+ SMCs and C6 S100A4+ SMCs, with relatively lower levels in other subpopulations. IRX1 exhibited minimal expression across all SMC subpopulations. KLF2 displayed higher expression in C4 ACTG2+ SMCs and was also detected in C0 SOCS3+ SMCs and C6 S100A4+ SMCs **(**Fig. 8E**)**.

Regarding AUC values **(**Fig. 8F**)**, EGR1 showed the highest in C0 SOCS3+ SMCs, followed by C4 ACTG2+ SMCs and C6 S100A4+ SMCs. NR2F2 had the highest AUC in C1 CD36+ SMCs, followed by C5 IGF2+ SMCs. HEY2 exhibited relatively high AUC values in C0 SOCS3+ SMCs, C4 ACTG2+ SMCs, C6 S100A4+ SMCs, and C2 SORBS2+ SMCs. IRX1 had the highest AUC in C8 EGR1+ SMCs, followed by C3 RGS5+ SMCs and C7 C12orf75+ SMCs. KLF2 showed relatively high AUC values in C4 ACTG2+ SMCs, C6 S100A4+ SMCs, and C0 SOCS3+ SMCs.

### Identification of TF regulatory modules in cardiomyopathy SMCs

**Fig. 9:**
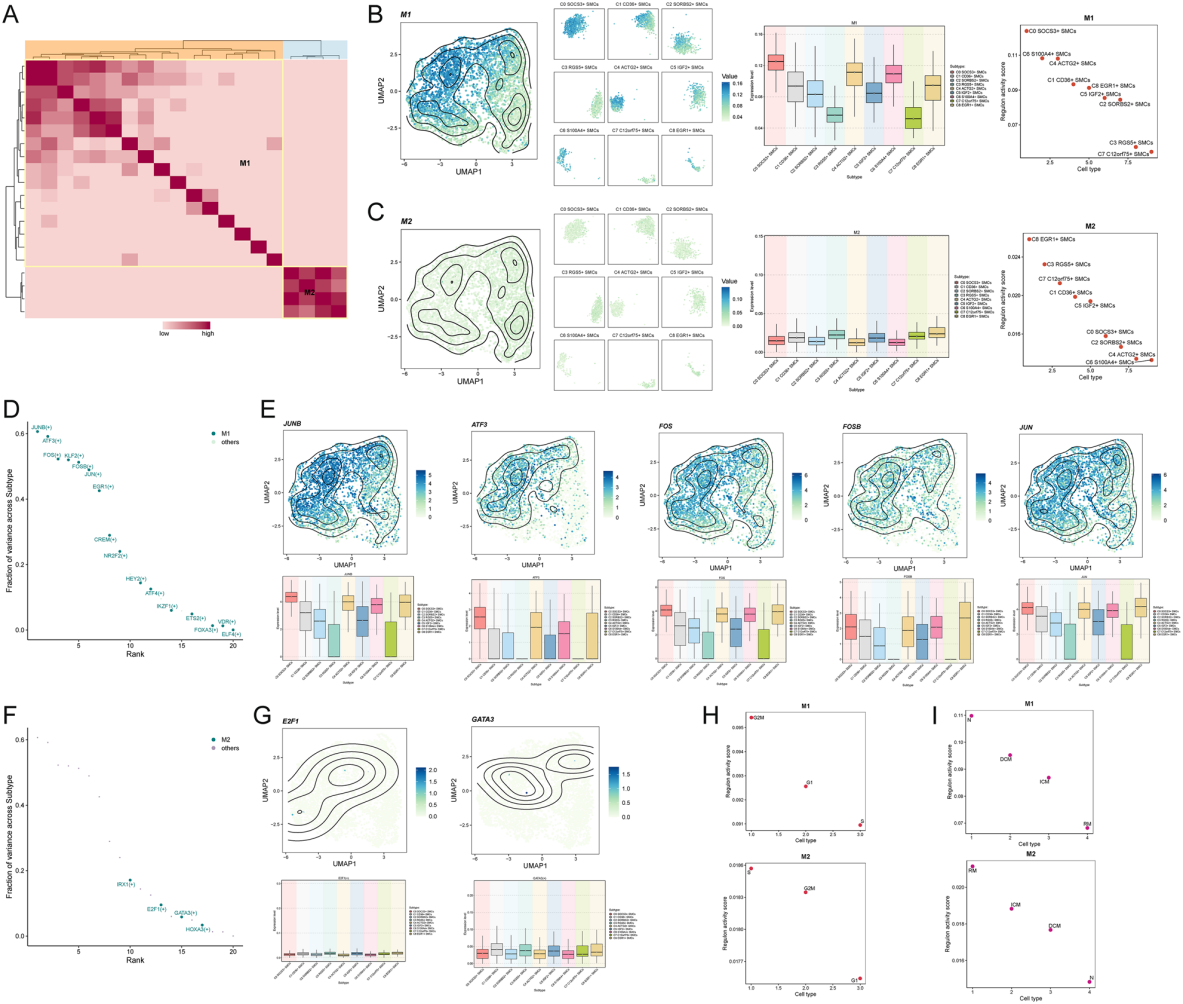
Identification of transcription factor regulatory modules in cardiomyopathy smooth muscle cells (**A**) The heatmap displayed the identification of regulatory submodules in smooth muscle cell subpopulations associated with cardiomyopathy based on SCENIC regulatory rules modules and AUCell scores similarity. Eventually, two rule-based submodules were identified. (**B, C**) UMAP plots and faceted plots showed the distribution of AUC scores of two rule-based submodules (M1, M2) and their distribution across various smooth muscle cell subpopulations (left). Box plots intuitively compared the expression levels of M1 and M2 in each smooth muscle cell subpopulation (middle). Scatter plots provided a better understanding of the expression score rankings of M1 and M2 in each smooth muscle cell subpopulation (right). (**D**) Scatter plot displayed the ranking of different TFs in M1 based on their variation scores. (**E**) Visualization of the 5 TFs with high variation scores in M1. UMAP plots illustrated the expression distribution of JUNB, ATF3, FOS, FOSB, and JUN, while box plots compared their expression differences among the nine smooth muscle cell subpopulations. (**F**) Scatter plot depicted the variation scores of different TFs in M2. (**G**) Visualization of the 2 TFs with high ranking in M2. UMAP plots respectively showed the expression distribution of these TFs, while box plots compared their expression differences among the nine smooth muscle cell subpopulations. (**H**) Scatter plots showed the ranking of the main cell cycle phases in M1 and M2. (**I**) Scatter plots showed the ranking of the groups to which cells belonged in M1 and M2.

Using SCENIC, we identified regulatory modules and rules in SMC subpopulations associated with cardiomyopathy. Based on AUCell scores’ similarity, we categorized these rules into two main modules, M1 and M2 **(**Fig. 9A**)**. Mapping the average activity scores of each module onto the UMAP plot revealed distinct distributions across various SMC subpopulations, as depicted in the facet plot. From the boxplot analysis, M1 prominently featured C0 SOCS3+ SMCs, C4 ACTG2+ SMCs, and C6 S100A4+ SMCs, while M2 included fewer subpopulations, with slightly higher proportions observed in C8 EGR1+ SMCs, C3 RGS5+ SMCs, and C5 IGF2+ SMCs.

We ranked the SMC subpopulations within M1 and M2 based on their transcription factor activity scores. In M1, the ranking was C0 SOCS3+ SMCs > C6 S100A4+ SMCs > C4 ACTG2+ SMCs > C1 CD36+ SMCs > C8 EGR1+ SMCs > C5 IGF2+ SMCs > C2 SORBS2+ SMCs > C3 RGS5+ SMCs > C7 C12orf75+ SMCs. In M2, the ranking was C8 EGR1+ SMCs > C3 RGS5+ SMCs > C7 C12orf75+ SMCs > C1 CD36+ SMCs > C5 IGF2+ SMCs > C0 SOCS3+ SMCs > C2 SORBS2+ SMCs > C4 ACTG2+ SMCs > C6 S100A4+ SMCs **(**Fig. 9B and C**)**.

We visualized the TFs in M1 and M2. In M1, TFs with high variance scores included JUNB, ATF3, FOS, KLF2, FOSB, JUN, and EGR1 **(**Fig. 9D**)**. In contrast, TFs in M2 generally exhibited lower variance scores, although IRX1, E2F1, and GATA3 showed relatively higher levels of activity **(**Fig. 9F**)**.

We depicted the expression levels and density distribution of JUNB, ATF3, FOS, FOSB, and JUN in M1 on the UMAP plot. From the boxplot, we observed that JUNB was highly expressed in C0 SOCS3+ SMCs, C4 ACTG2+ SMCs, C8 EGR1+ SMCs, and C6 S100A4+ SMCs; ATF3 was highly expressed in C0 SOCS3+ SMCs, C4 ACTG2+ SMCs, and C6 S100A4+ SMCs; FOS showed high expression in C0 SOCS3+ SMCs, C4 ACTG2+ SMCs, C8 EGR1+ SMCs, and C6 S100A4+ SMCs; FOSB exhibited high expression in C8 EGR1+ SMCs, C0 SOCS3+ SMCs, C6 S100A4+ SMCs, and C4 ACTG2+ SMCs; JUN displayed high expression in C8 EGR1+ SMCs, C0 SOCS3+ SMCs, C4 ACTG2+ SMCs, and C6 S100A4+ SMCs **(**Fig. 9E**)**.

Similarly, we mapped the AUC value distribution of E2F1 and GATA3 onto the UMAP plot. From the boxplot, E2F1 showed lower AUC values across all SMC subpopulations, whereas GATA3 exhibited slightly higher values in C1 CD36+ SMCs, C3 RGS5+ SMCs, and C5 IGF2+ SMCs **(**Fig. 9G**)**.

Furthermore, we ranked cell cycle phases and groups based on transcription factor activity scores **(**Fig. 9H and I**)**. In M1, the order was G2M phase > G1 phase > S phase, N > DCM > ICM > RM. In M2, the order was S phase > G2M phase > G1 phase, RM > ICM > DCM > N.

### Lowering S100A4 suppresses HA-VSMC cell proliferation

**Fig. 10:**
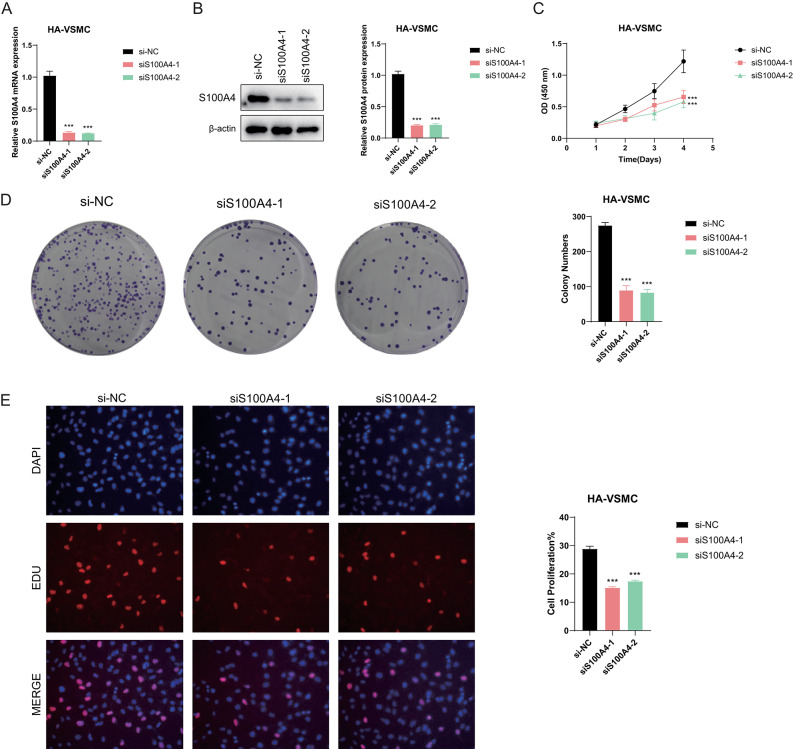
Knockdown of S100A4 suppresses the proliferation ability of HA-VSMCs (**A**) Relative expression of S100A4 in Si-S100A4 knockdown HA-VSMCs. (**B**) Differential expression of S100A4 protein in HA-VSMCs with Si-S100A4 knockdown. (**C**) The CCK-8 assay revealed a significant decrease in cell viability following S100A4 knockdown. (**D**) Colony formation assay showed a markedly reduced number of cell colonies in cells with depleted S100A4 expression compared to the NC group. (**E**) Edu staining assay demonstrated that downregulation of S100A4 significantly inhibited HA-VSMC proliferation relative to the NC group. Statistical significance was indicated as *P < 0.01, **P < 0.001, ***P < 0.0001, and ****P < 0.00001.

To elucidate the role of S100A4 in myocardial disease, particularly its impact on smooth muscle cell behavior in ischemic cardiomyopathy (ICM), we conducted rigorous in vitro experiments. Initially, we quantified S100A4 expression levels using qRT-PCR after 24-h transfection with siRNA in the HA-VSMC cell line, confirming effective S100A4 knockdown **(**Fig. 10A**)**. Next, we verified the relative protein expression after S100A4 knockdown, and it turned out that S100A4 protein expression was significantly reduced **(**Fig. 10B**)**. Subsequently, CCK8 assays unequivocally demonstrated a significant decrease in HA-VSMC viability following S100A4 knockdown (P < 0.001) **(**Fig. 10C**)**. Colony formation assays further supported these findings, showing a marked reduction in colony counts in both S100A4 knockdown groups compared to the non-knockdown control group (NC) **(**Fig. 10D**)**. The observed slower rate of colony formation in cells with S100A4 knockdown strongly suggests the crucial role of S100A4 in promoting HA-VSMC proliferation. Furthermore, results from EdU staining experiments provided compelling evidence that downregulation of S100A4 expression hindered the proliferation of HA-VSMC cells relative to the NC group **(**Fig. 10E**)**.

### Knocking down S100A4 suppresses cell migration and invasion of HA-VSMC and induces apoptosis

**Fig. 11:**
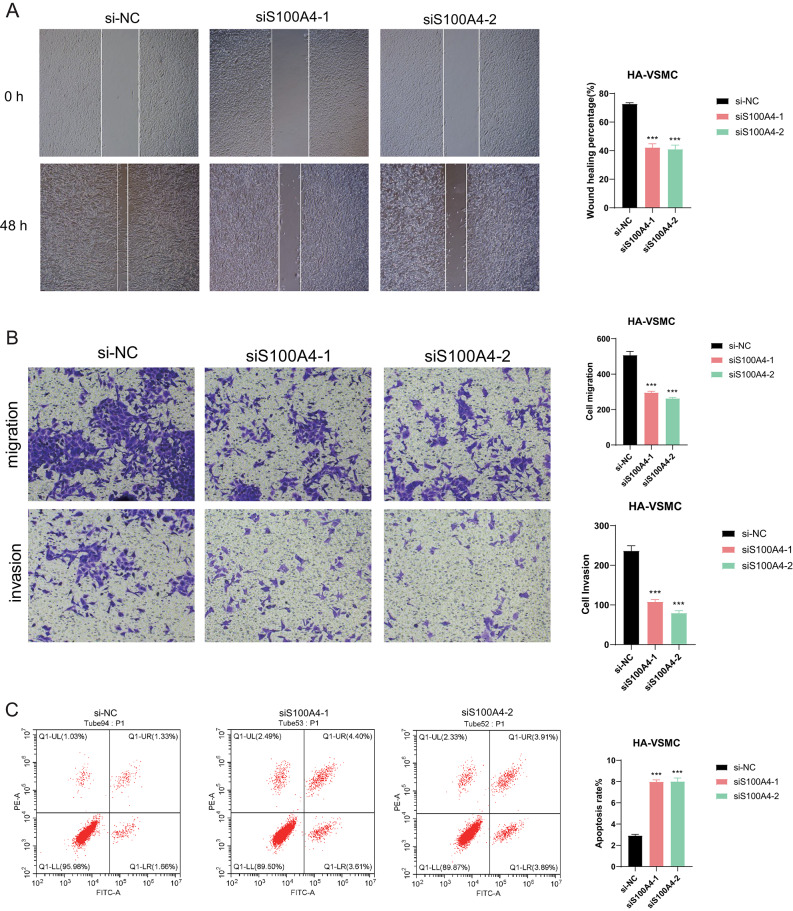
Knockdown of S100A4 suppresses the migration and invasion abilities of HA-VSMCs and induces cell apoptosis (**A**) The scratch wound healing assay revealed that decreased expression of S100A4 significantly impaired the wound healing rate. (**B**) The Transwell assay demonstrated that downregulation of S100A4 markedly reduced the migration and invasion capabilities of HA-VSMCs. (**C**) Annexin V-FITC/PI double staining flow cytometry assay showed a substantial increase in induced cell apoptosis following S100A4 knockdown compared to the NC group. Statistical significance was indicated as *P < 0.01, **P < 0.001, ***P < 0.0001, and ****P < 0.00001.

Subsequent findings from scratch wound healing assays revealed a significant reduction in the rate of wound closure following the downregulation of S100A4 expression **(**Fig. 11A**)**. Transwell assays further demonstrated a notable decrease in both the migration and invasion capabilities of HA-VSMC cells upon S100A4 knockdown **(**Fig. 11B**)**. Moreover, apoptosis in HA-VSMC was assessed using Annexin V-FITC/PI staining followed by flow cytometry analysis. As illustrated in Fig. 11C, knocking down S100A4 led to an increased proportion of apoptotic HA-VSMC cells. To ensure the accuracy and consistency of our results, all assays were conducted using a single smooth muscle cell line (HA-VSMC), and the data are presented as the mean standard deviation of independent experiments. Statistical significance is indicated as *P < 0.05, **P < 0.01, ***P < 0.001.

## Discussion

Cardiomyopathies encompass a broad spectrum of disorders that significantly impact the intricate functioning of the heart^62^. The etiology of these conditions is widely recognized to involve complex interactions between genetic predisposition and environmental factors^63^. These ailments are typically classified into distinct types, such as DCM, ICM and RM. Scientific investigation has validated a close association between the intricate mechanisms of ferroptosis—a regulated form of cell death dependent on disruptions in iron metabolism—and various cardiomyopathies, particularly ICM and DCM. Specifically, under conditions like coronary atherosclerosis-induced ischemia, cardiomyocytes endure damage due to excessive iron accumulation^64^. Moreover, the occurrence of ferroptosis exacerbates oxidative stress, leading to detrimental effects such as compromised integrity of cardiomyocyte membranes and dysfunction of cardiovascular endothelial cells^65^. In a murine model of myocardial ischemia, G. Drossos et al. observed a significant decrease in the expression of FTH, impairing cardiomyocytes’ ability to sequester free iron and resulting in increased oxidative stress and, in severe cases, cell death^66^. Thus, a plausible link emerges between ferroptosis, oxidative stress, and their collaborative roles in shaping the progression of cardiomyopathies. Furthermore, ferroptosis in VSMCs correlates positively with the advancement of atherosclerosis^67^. Therefore, our primary research focuses on VSMCs as a pivotal area of investigation. We aim to explore key smooth muscle cell subsets and hope to find and validate possible target genes. To achieve this goal, we rigorously analyzed scRNA-seq data to identify diverse SMC subpopulations implicated in cardiomyopathies, employing advanced visualization techniques to unravel their intricate features.

To dissect the nuanced variations within SMC subpopulations with precision, we meticulously categorized 4,148 high-quality SMCs into nine distinct clusters. Each cluster was distinguished by a prominent marker gene: C0 SOCS3+ SMCs, C1 CD36+ SMCs, C2 SORBS2+ SMCs, C3 RGS5+ SMCs, C4 ACTG2+ SMCs, C5 IGF2+ SMCs, C6 S100A4+ SMCs, C7 C12orf75+ SMCs, and C8 EGR1+ SMCs. Notably, S100A4, a crucial regulator of vascular remodeling, plays a pivotal role in promoting angiogenesis and inducing oxidative stress-related damage in vascular endothelial cells^68–70^. However, research on the involvement of S100A4 in SMCs is currently limited, with few studies establishing a direct correlation between S100A4 levels and cardiovascular risk^71^. In addition to affecting ECs, the secretion of inflammatory cytokines and angiogenic growth factors influences both ECs and SMCs, impacting the development of atherosclerosis. The proliferation of SMCs, crucial in atherosclerosis progression, ultimately contributes to ICM^72–74^. Among the genes highly expressed in and closely associated with C6 S100A4+ SMCs are IER2, C11orf96, ZFP36, PPP1R15A, BTG2, SERTAD1, SOCS3, and EGR. We were pleased that several of these eight genes have been previously identified as pro-inflammatory genes, which has a non-negligible link with the occurrence and development of cardiomyopathy that we are studying^75,76^. Among these, research on IER2 in the context of ICM is scarce. However, studies have found that IER2 is upregulated in adipose stromal cells after vitamin C treatment and may participate in a novel nutrient-responsive gene pathway in adipose tissue^77^, potentially relevant to early atherosclerotic plaque formation. This warrants further investigation into its role in the pathogenesis of ICM. Research on C11orf96 is also extremely limited, with only a few studies reporting its upregulation in lung cancer tumor tissues^78,79^. ZFP36, which is expressed in atherosclerotic lesions^80^, may have a potential connection to ICM. What’s more, ZFP36 is a known pro-inflammatory gene involved in the regulation of inflammatory responses. It promotes the expression of a variety of inflammatory factors by regulating the stability of mRNA^81^. Sparse studies have linked BTG2 to cardiac hypertrophy^82^. Ischemia-induced SOCS3 may promote apoptosis during ventricular remodeling, exacerbating myocardial necrosis and potentially leading to heart failure^83,84^. And SOCS3 is an important immunomodulatory factor, which regulates the inflammatory response by inhibiting the cytokine signaling pathway, but its effect has a negative feedback nature^85^. The EGR gene family includes multiple members, such as EGR1, which are usually activated when cells respond to various stimuli, such as inflammatory stimuli. EGR1 in particular is thought to play an important role in the pro-inflammatory response^86^. Furthermore, overexpression of EGR has been shown to induce cardiac hypertrophy and arrhythmias^87^. In contrast, PPP1R15A and SERTAD1 lack research evidence supporting their association with cardiomyopathy. Furthermore, our visualizations demonstrated the differences in the cell cycle across subpopulations. It is well established that an increase in cells in the G1 phase indicates cell cycle arrest^88^. Moreover, previous studies showed that knocking down S100A4 in other cell types led to cell cycle arrest^89^. However, whether knocking down S100A4 produced the same effect in SMCs lacked corresponding research evidence. To address this, we conducted in vitro experiments using a human aortic smooth muscle cell line. Following S100A4 knockdown, we observed reduced proliferation and migration of VSMCs, along with increased apoptosis. More importantly, our annotations revealed that S100A4 was the most strongly expressed marker gene in the C6 subpopulation, prompting us to designate S100A4 as the naming gene for this group. Overall, the cell cycle was only one aspect of evidence for cellular proliferative capacity. Other features examined in this study, such as stemness, metabolism, differentiation potential, and developmental trajectories, further supported the proliferative ability of these cells. Based on these findings, we identified the C6 subpopulation as having strong proliferative capacity and high S100A4 expression. We therefore hypothesized that S100A4 has stronger expression in cells without growth arrest. Taken together, we proposed that the C6 subpopulation exhibited significant proliferative capacity and functional importance. This led us to hypothesize a strong correlation between the C6 S100A4+ SMCs subpopulation and the pathogenesis as well as the adverse progression of cardiomyopathy. Therefore, we decided to focus future research on this intriguing C6 S100A4+ SMCs subpopulation.

Next, a comprehensive analysis of GO-BP has revealed that C6 S100A4+ SMCs are notably enriched in complex processes such as the regulation of neurogenesis, nervous system development, and circadian gene expression regulation. Importantly, ferroptosis—a critical component in neuronal cell demise and neurodegenerative diseases—emerges as an intriguing possibility for potential interconnections with C6 S100A4+ SMCs. These insights open up exciting avenues for further exploration into the role of S100A4 in SMCs and its implications in cardiovascular health and disease, suggesting novel therapeutic targets and mechanisms underlying cardiomyopathies.

Building on our earlier analysis, we have delved deeper into visualizing the cellular growth activity within C6 S100A4+ SMCs. Leveraging stemness scoring, which correlates closely with cell growth dynamics and intrinsic cellular characteristics^90^, our investigation unequivocally confirms that among various subgroups, C6 S100A4+ SMCs exhibit the highest degree of stemness, thereby substantiating our initial hypothesis. To further elucidate these findings, we investigated the expression patterns of five prominent stemness genes: EPAS1, CTNNB1, MYC, HIF1A, and NES. Notably, CTNNB1 plays a dual role in cardiac development, persisting in myocardial structures even amid cardiac mutations^91,92^. MYC is implicated in potential critical roles in heart failure^93^, while the stability of HIF1A is linked to metabolic shifts towards increased glucose utilization in cardiomyopathy and aging^94^. Thus, establishing potential associations between cardiomyopathies and these stemness genes is plausible. The heightened level of cellular stemness and robust cell growth observed in C6 S100A4+ SMCs suggest an equally active state of cellular metabolism. In this context, we assessed the prominence of metabolic pathways within this subgroup, identifying oxidative phosphorylation as the predominant metabolic process. Genetic anomalies affecting oxidative phosphorylation have been implicated in precipitating cardiomyopathies. A notable study by Lu et al.^95^ using a mouse model highlighted significant similarities in differentially expressed proteins between ICM and DCM hearts, primarily mediated through pathways associated with oxidative phosphorylation. This underscores the pivotal role of oxidative phosphorylation in heart failure pathogenesis.

In summary, our comprehensive findings suggest that C6 S100A4+ SMCs may hold relevance to cardiomyopathies, particularly concerning cellular growth, stemness, and metabolic processes. These insights pave the way for further exploration into novel therapeutic targets and mechanisms underlying cardiomyopathies, potentially offering new avenues for intervention and treatment strategies.

Subsequently, we proceeded to assign scores to all SMC subpopulations using a gene set library specifically associated with ferroptosis. Notably, C6 S100A4+ SMCs exhibited relatively elevated scores, effectively corroborating our initial hypothesis. In the subsequent assessment, we evaluated all subpopulations against four distinct pathways linked to oxidative stress. In the first three pathways, which involve positive regulation or response to oxidative stress, C6 S100A4+ SMCs consistently attained higher scores compared to other subpopulations. Conversely, in the pathway associated with the negative regulation of the oxidative stress response, C6 S100A4+ SMCs recorded notably low scores. This compellingly reinforces our contention that C6 S100A4+ SMCs represent a subpopulation highly likely to be involved in ferroptosis and susceptible to oxidative stress.

The degree of cell differentiation is a crucial indicator reflecting ongoing cellular growth and proliferation activity. To validate the characteristics of C6 S100A4+ SMCs, we employed trajectory inference using the Slingshot algorithm. Remarkably, our results indicate that C6 S100A4+ SMCs predominantly occupy early stages across all three lineages studied. This compellingly demonstrates that the majority of C6 S100A4+ SMCs exist in a state of active growth, marked by heightened mRNA and gene expression. Consequently, these findings suggest a profound potential for these SMCs to significantly contribute to the process of SMC proliferation.

Furthermore, the GO-BP analysis of genes showing differential expression at the stage where C6 S100A4+ SMCs are located revealed significant enrichment in terms related to radiation, mRNA, nuclear transcription, proliferation, stress, transcription, muscle, death, and destabilization. Most of these terms are directly associated with cell growth, proliferation, apoptosis, and stress responses. Thus, this thorough analysis provides strong evidence that substantiates our initial hypothesis.

Based on these findings, it can be inferred that C6 S100A4+ SMCs are closely linked to the exacerbation of cardiomyopathy, potentially contributing to its progression. Adhering to rigorous research principles, we utilized CytoTRACE to evaluate cellular stemness, revealing that C6 S100A4+ SMCs exhibit the highest stemness level and the lowest degree of differentiation. Additionally, Monocle analysis further supports these observations by showing that the majority of C6 S100A4+ SMCs are positioned early in the developmental trajectory, with only a small fraction in the terminal segment. Consistency with results from Slingshot and stemness scoring provides further confirmation that C6 S100A4+ SMCs represent a subset of SMCs characterized by limited differentiation but heightened activity in growth and proliferation.

Moreover, extensive research has focused on exploring the intricate interactions between SMCs and ECs^96^. Endothelial dysfunction, a well-established pathological condition affecting ECs, is closely associated with various diseases such as hypertension, atherosclerosis, and diabetes. These conditions often involve changes in vascular reactivity and remodeling of the vascular wall, which particularly affect SMCs. Consequently, these phenomena could significantly influence the development and progression of disorders like ICM^97,98^. Therefore, it is hypothesized that the interplay between SMCs and ECs may play a pivotal role in the pathogenesis of cardiomyopathies, although the precise mechanisms remain incompletely understood.

Through the application of CellChat analysis, a compelling finding emerged: C6 S100A4+ SMCs function as signal transmitters, while ECs act as signal receivers, forming a ligand-receptor pair identified as PTN-NCL. This interaction has been visually confirmed and validated, providing concrete evidence of its occurrence. Notably, PTN expression on C6 S100A4+ SMCs has been shown to enhance EC migration, potentially leading to an increased local EC population^99,100^. NCL, a multifunctional protein, plays a dynamic role in the proliferation of VSMCs and acts as a mediator for PTN’s stimulatory effects. Furthermore, blocking or downregulating NCL on EC surfaces effectively inhibits EC migration, impairs capillary tube formation, and promotes EC apoptosis^101,102^. Specifically, PTN enhances the proliferation and migration of ECs, while NCL boosts the proliferation of C6 S100A4+ SMCs and inhibits EC apoptosis. The synergistic actions of PTN and NCL mutually reinforce their effects, collectively exerting a significant impact on the initiation and progression of cardiomyopathies.

To explore the key TFs in specific subpopulations of SMCs implicated in cardiomyopathies, we conducted a gene regulatory network analysis. Initially, our investigation identified KLF2 as the most dynamically active TF in C6 S100A4+ SMCs. Activated KLF2 regulates critical functions in ECs, including proliferation, migration, and inflammation, playing a protective role in maintaining EC integrity and health^103,104^. Consequently, C6 S100A4+ SMCs may influence cardiomyopathies by promoting EC proliferation and migration through heightened KLF2 expression.

Using data from the Comprehensive Sequence-based Investigation (CSI), we identified two primary modules, M1 and M2, within SMC subpopulations associated with cardiomyopathies. Importantly, C6 S100A4+ SMCs predominantly belong to module M1. Subsequently, we examined TFs with the highest rankings in M1. JUNB, ATF3, FOS, FOSB, and JUN were found to exhibit elevated expression levels in C6 S100A4 + SMCs. ATF3, in particular, shows heightened expression specifically in myocardial tissue affected by DCM, although its precise role in cardiac physiology remains debated^105,106^. Emerging evidence suggests that inhibiting FOS may potentially mitigate cardiac fibrosis and inflammation^107^. Additionally, there is a documented association between fibrosis and the activation of TFs FOSB and JUNB^108^. Cardiac fibrosis is a detrimental outcome associated with cardiomyopathies, particularly in cases linked to diabetes-triggered DCM. Hence, it is plausible that the release of TFs FOS, FOSB, and JUNB by C6 S100A4+ SMCs contributes to exacerbating cardiomyopathies, potentially through the induction of cardiac fibrosis and inflammation.

Finally, as mentioned when selecting the C6 subtype, we conducted in vitro experiments to determine whether the knockdown of S100A4 similarly blocks cell growth and promotes apoptosis in SMCs, with the aim of exploring its role in cardiomyopathy, particularly in ICM. Initially, using siRNA-mediated knockdown, we observed a significant decrease in the viability of HA-VSMCs after S100A4 downregulation, as confirmed by CCK8 assays. Further studies involving colony formation and Edu staining experiments confirmed that S100A4 positively promotes the proliferation of HA-VSMCs. Furthermore, using scratch wound healing and Transwell assays, we determined that knockdown of S100A4 significantly inhibited HA-VSMCs migration and invasion. Flow cytometry analysis showed that the proportion of apoptotic HA-VSMCs increased after S100A4 knockdown, indicating that S100A4 knockdown exhibits apoptosis-promoting properties. Based on these findings, we concluded that knockdown of S100A4 in C6 S100A4+ SMCs inhibits SMCs proliferation, migration and invasion and promotes apoptosis. This may serve as indirect evidence that S100A4 may have a necessary link with the proliferation of SMCs, leading to fibrosis and pathological ventricular remodeling, which impairs cardiac function and ultimately accelerates the progression of cardiomyopathy^109,110^. Of course, further studies are needed to prove this. In addition, we will also conduct further experiments to verify the specific regulatory effect of S100A4 on the cell cycle process.

## Conclusion

Based on our comprehensive single-cell characterization of SMC subsets in cardiomyopathy, as well as analysis of relevant transcriptional regulatory networks and in vitro experimental validation, our study concludes that C6 S100A4+ SMCs exhibit increased sensitivity to adverse events associated with cardiomyopathy. This sensitivity may be influenced by processes involved in ferroptosis, oxidative phosphorylation, and inflammatory response, as C6 subgroup has higher such scores, but whether it is really affected by them needs further research to prove.

In addition, C6 S100A4+ SMCs had higher scores for genes related to ferroptosis, oxidative stress and inflammation, which may indicate a new potential therapeutic approach for treating cardiomyopathy and preventing heart failure. The identification of PTN-NCL protein receptor pairs between C6 S100A4+ SMCs and ECs also provides a possible potential target for therapeutic intervention in cardiomyopathy. In addition, stem-like genes such as CTNNB1, MYC, and HIF1A may also attenuate heart failure after cardiomyopathy. KLF2, a regulator identified in our study, has been shown by several studies to inhibit EC growth, proliferation and migration, so it also shows potential as a therapeutic target for the prevention of cardiomyopathy. The transcription factors FOS, FOSB, and JUNB may help prevent adverse events such as cardiac fibrosis in cardiomyopathy.

In summary, these findings provide new insights into the future prevention, treatment, and management of cardiomyopathies. However, further refinement and advancement of our research are necessary to fully harness the therapeutic potential of these discoveries.

## Study limitations

We must acknowledge certain unavoidable limitations that may introduce some degree of error. Some of these limitations include, for example, the possibility that patient-derived samples may be affected by the administration of positive inotropic drugs, which may affect signaling mechanisms and differential transcription patterns. This may lead to small differences in the strength and frequency of communication signals between cells, as well as changes in transcriptional patterns within subpopulations. In addition, C6 subpopulation is derived from two ventricles, and it has been reported that in different pathogenesis, cells from the two ventricles respond to various signals differently and tend to adopt different functions, and each ventricle is unique. Therefore, the heterogeneity between the two ventricles may play a role in the characterization of C6 subgroup. Therefore, fully considering these potential errors and striving to minimize their effects will remain an important aspect of our work in our future studies. Further in vitro studies on ferroptosis, oxidative stress, PTN-NCL signaling pathway, and related ligand receptor proteins and genes will be performed to obtain more important and interesting findings.

## Supplementary Information

Supplementary Information 1.

Supplementary Information 2.

Supplementary Information 3.

## Data Availability

Data is provided within the manuscript. The original data and other data related to this study are available upon request from the corresponding author (email: 71,000,799@sdutcm.edu.cn).
