# Supplementary material for: Identification of a key smooth muscle cell subset driving ischemic cardiomyopathy progression through single-cell RNA sequencing
Source: Sci Rep. 2025 Jul 27;15:27331. doi: 10.1038/s41598-025-09928-6 (PMC12301475; doi:10.1038/s41598-025-09928-6)
Supplement: Supplementary file 1 — Supplementary Information 1. [file 41598_2025_9928_MOESM1_ESM.docx]

**Figure S1**


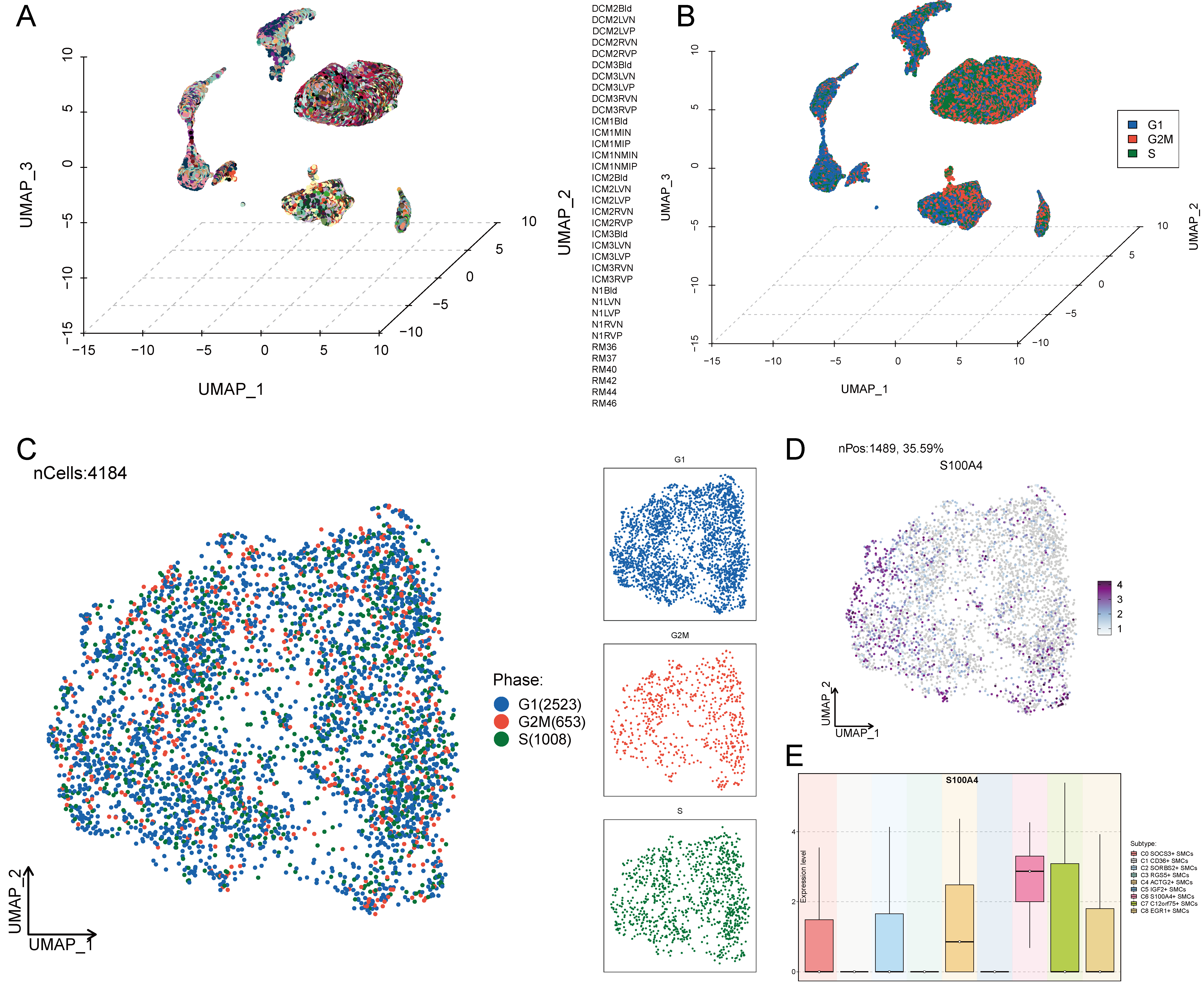


**Figure S1**: **Cell origin, cell cycle status, and S100A4 expression characteristics of each SMC subpopulation** (A) The UMAP plot showed the sample sources of all high-quality cells. (B) The UMAP plot showed the cell cycle status of all high-quality cells. (C) The UMAP plot showed the cell cycle distribution of all SMC subpopulations. (D) Differential expression of S100A4 was observed across all subpopulations. (E) S100A4 was highly expressed in the C6 subpopulation.
