# Supplementary material for: Identification of a key smooth muscle cell subset driving ischemic cardiomyopathy progression through single-cell RNA sequencing
Source: Sci Rep. 2025 Jul 27;15:27331. doi: 10.1038/s41598-025-09928-6 (PMC12301475; doi:10.1038/s41598-025-09928-6)
Supplement: Supplementary file 2 — Supplementary Information 2. [file 41598_2025_9928_MOESM2_ESM.pdf]

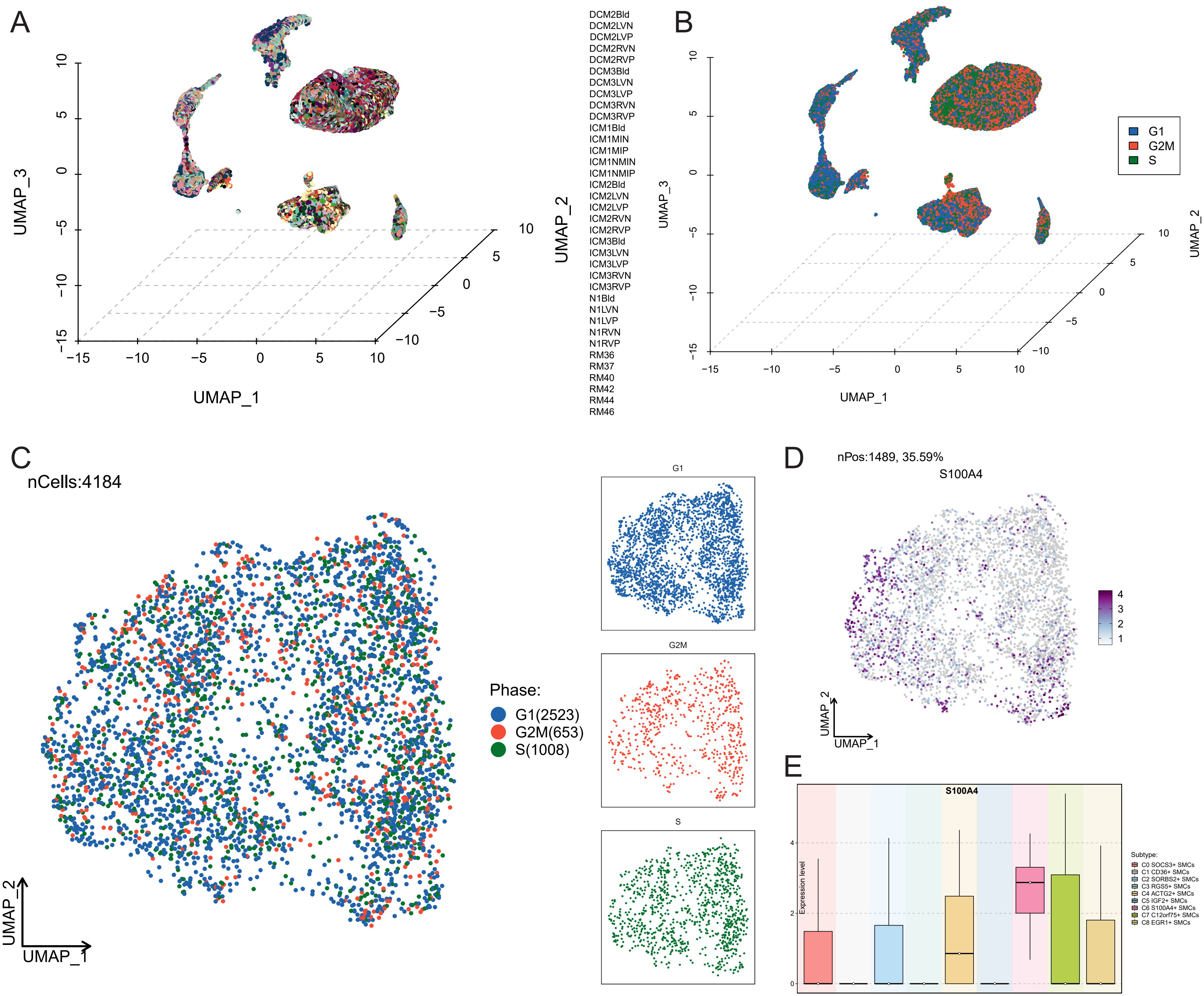
A
UMAP_3
UMAP_1
UMAP_2
DCM2Bld
DCM2LVN
DCM2LVP
DCM2RVN
DCM2RVP
DCM3Bld
DCM3LVN
DCM3LVP
DCM3RVN
DCM3RVP
ICM1Bld
ICM1MIN
ICM1MIP
ICM1NMIN
ICM1NMIP
ICM2Bld
ICM2LVN
ICM2LVP
ICM2RVN
ICM2RVP
ICM3Bld
ICM3LVN
ICM3LVP
ICM3RVN
ICM3RVP
N1Bld
N1LVN
N1LVP
N1RVN
N1RVP
RM36
RM37
RM40
RM42
RM44
RM46
B
G1
G2M
S
C
nCells:4184
Phase:
G1(2523)
G2M(653)
S(1008)
G1
G2M
S
D
nPos:1489, 35.59%
S100A4
E
S100A4
Expression level
Subtype:
C0 SOCS3+ SMCs
C1 CD36+ SMCs
C2 SORBS2+ SMCs
C3 RGS5+ SMCs
C4 ACTG2+ SMCs
C5 IGF2+ SMCs
C6 S100A4+ SMCs
C7 C12orf75+ SMCs
C8 EGR1+ SMCs
