# Supplementary material for: Identification of a key smooth muscle cell subset driving ischemic cardiomyopathy progression through single-cell RNA sequencing
Source: Sci Rep. 2025 Jul 27;15:27331. doi: 10.1038/s41598-025-09928-6 (PMC12301475; doi:10.1038/s41598-025-09928-6)
Supplement: Supplementary file 3 — Supplementary Information 3. [file 41598_2025_9928_MOESM3_ESM.pdf]

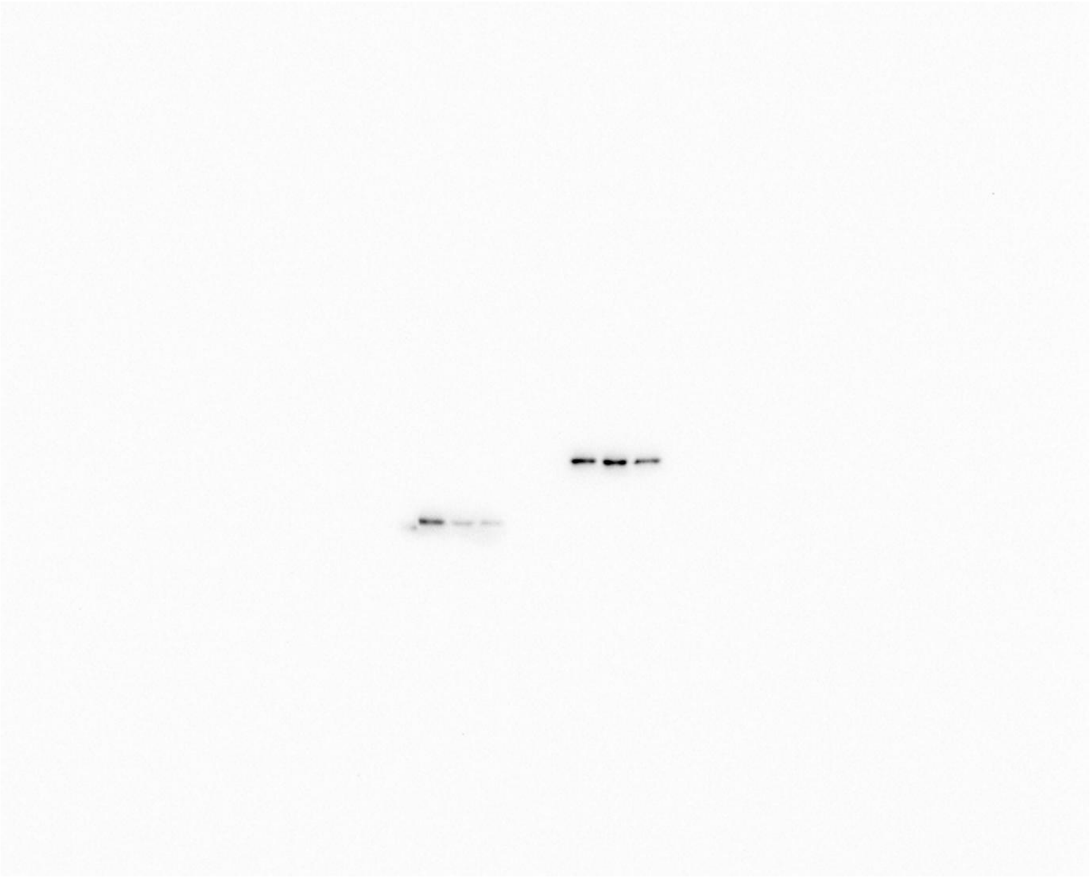

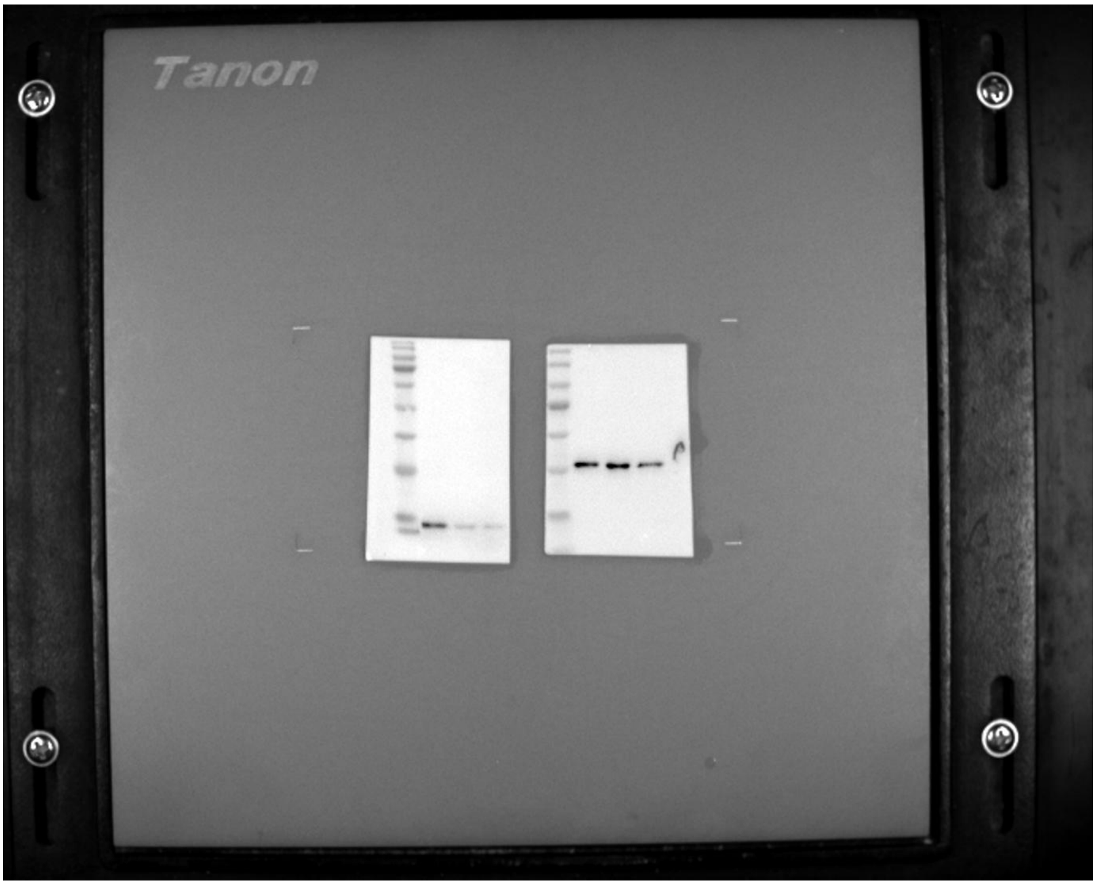

Biological repeat 1 (S100A4 and β-actin)

Left：S100A4
Right：β-actin

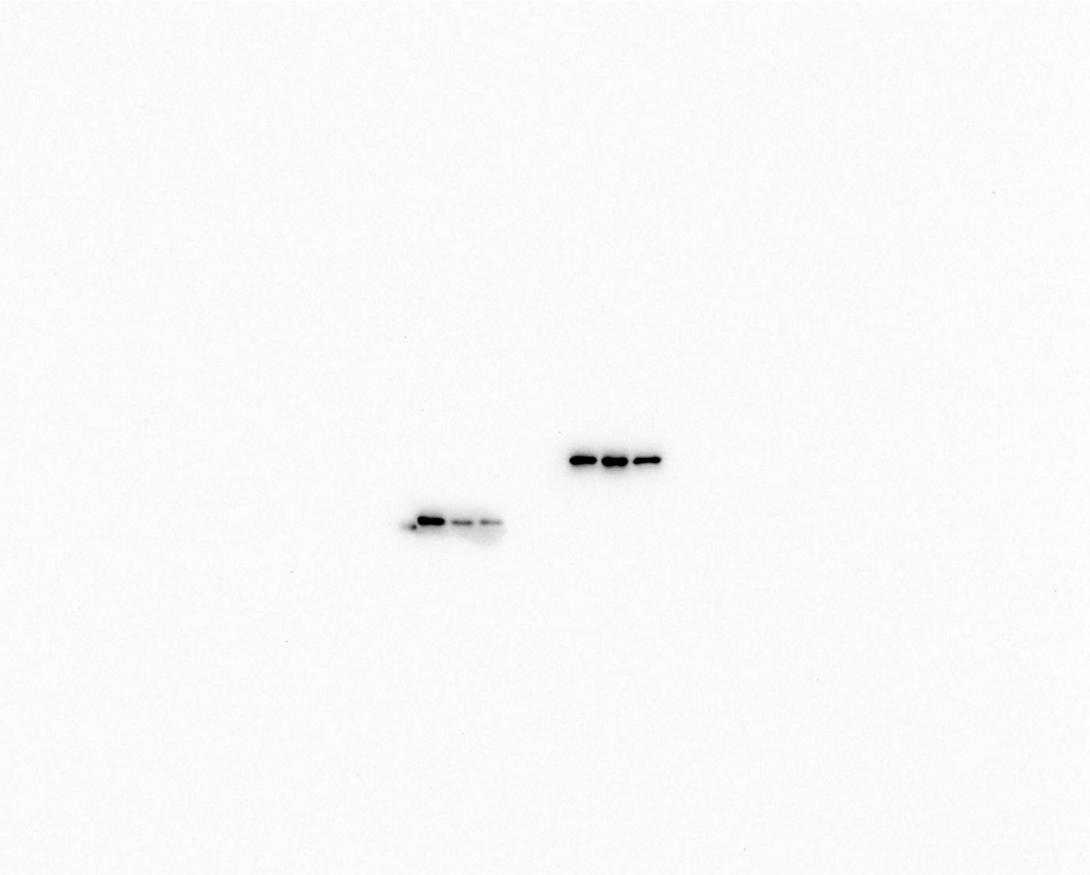

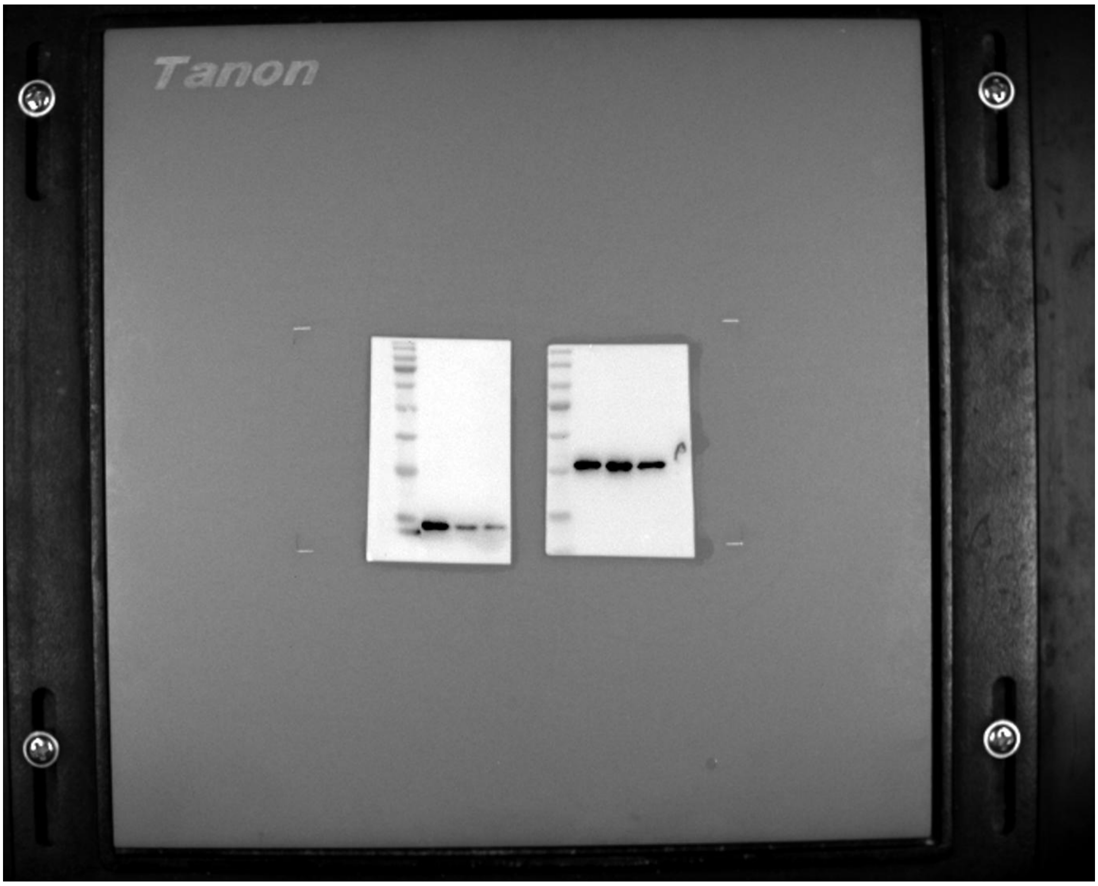

Biological repeat 2 (S100A4 and β-actin)

The original blots used in main figure

Left：S100A4
Right：β-actin

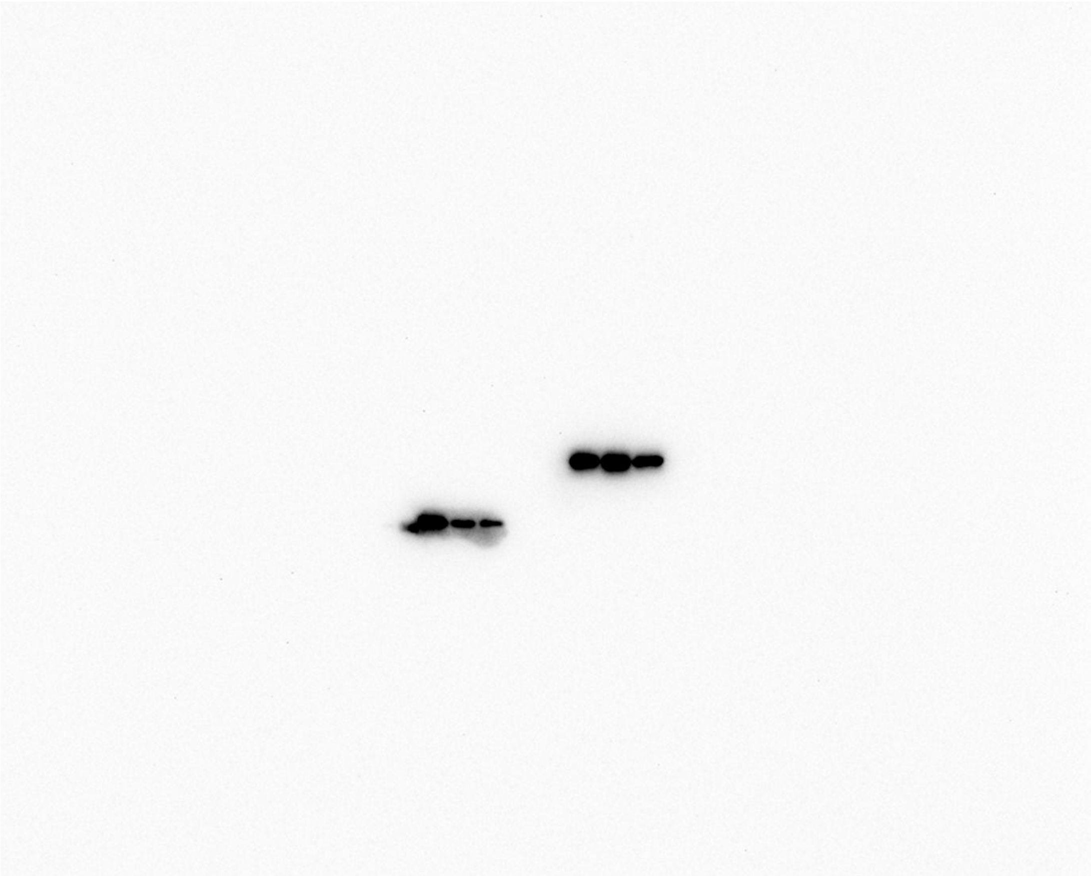

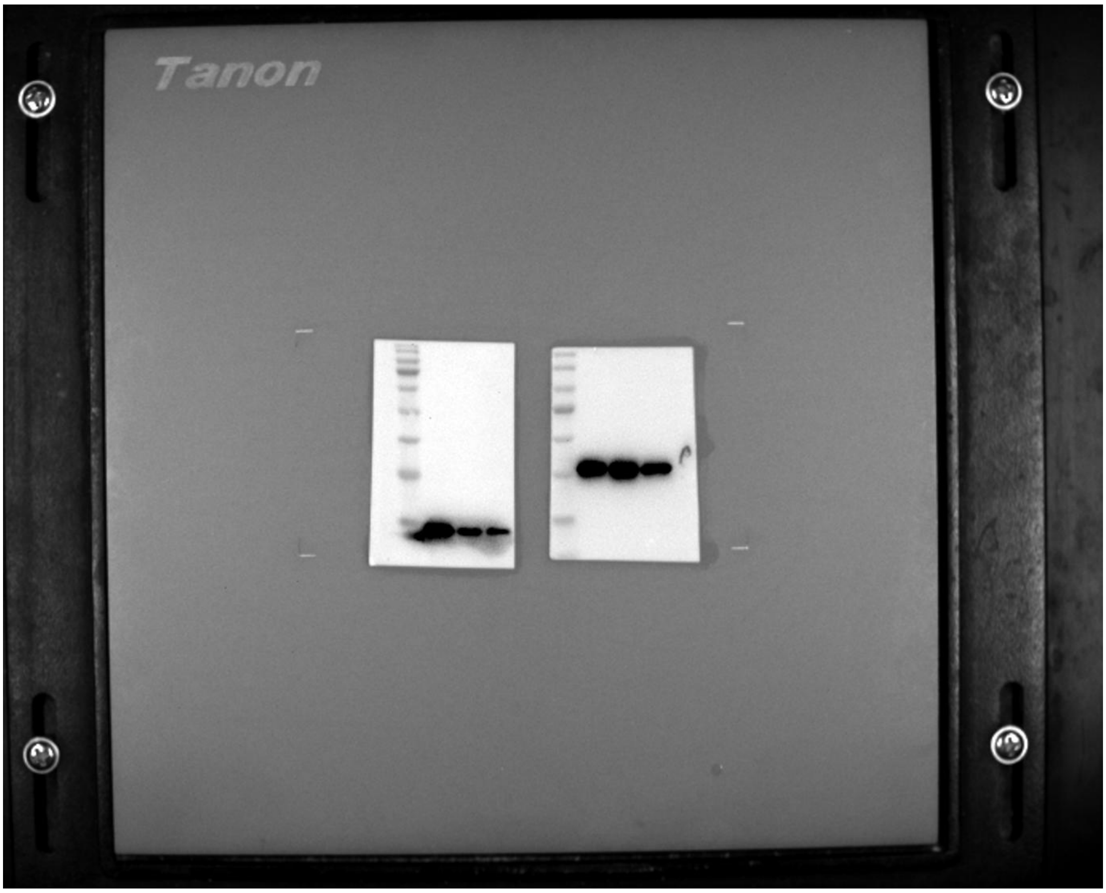

Biological repeat 3 (S100A4 and β-actin)

Left ：S100A4
Right ：β-actin
